# Evaluation of Filter Types for Trace Element Analysis in Brake Wear PM_10_: Analytical Challenges and Recommendations

**DOI:** 10.3390/molecules30244816

**Published:** 2025-12-18

**Authors:** Aleandro Diana, Mery Malandrino, Riccardo Cecire, Paolo Inaudi, Agnese Giacomino, Ornella Abollino, Agusti Sin, Stefano Bertinetti

**Affiliations:** 1 Department of Chemistry, University of Turin, 10125 Turin, Italy; aleandro.diana@unito.it (A.D.); riccardo.cecire@unito.it (R.C.); 2 UniTo-ITT JointLab, 10135 Turin, Italy; agusti.sin@itt.com; 3 Department of Agricultural, Forest and Food Sciences, University of Turin, 10095 Grugliasco, Italy; 4 Department of Drug Science and Technology, University of Turin, 10125 Turin, Italy; paolo.inaudi@unito.it (P.I.); agnese.giacomino@unito.it (A.G.); ornella.abollino@unito.it (O.A.); 5 ITT Friction Technologies, 12032 Barge, Italy

**Keywords:** metals, particulate matter, brakes, brake wear particles, ICP analysis

## Abstract

Accurate analysis of trace elements in particulate matter (PM) emitted by brake systems critically depends on the filter selection and handling processes, which can significantly impact analytical results due to contamination and elemental interference from filter elemental composition. This study systematically evaluated two widely used filter types, EMFAB (borosilicate glass microfiber reinforced with PTFE) and Teflon (PTFE), for their suitability in the trace element determination of brake-wear PM_10_ collected using a tribometer set-up. A total of twenty-three PM_10_ samples were analyzed, encompassing two different friction materials, to thoroughly assess the performance and analytical implications of each filter type. Filters were tested for their chemical background, handling practicality and potential contamination risk through extensive elemental analysis by inductively coupled plasma–optical emission spectrometry (ICP-OES) and inductively coupled plasma-mass spectrometry (ICP-MS). Additionally, morphological characterization of both filter types was conducted via scanning electron microscopy (SEM) coupled with energy-dispersive X-ray spectroscopy (EDS) to elucidate structural features affecting particle capture and subsequent analytical performance. Significant differences emerged between the two filters regarding elemental interferences: EMFAB filters exhibited substantial background contribution, particularly for alkali and alkaline earth metals (Ca, Na, Mg and K), complicating accurate quantification at trace levels. Conversely, Teflon filters demonstrated considerably lower background but required careful manipulation due to their structural fragility and the necessity to remove supporting rings, potentially introducing analytical variability. Statistical analysis confirmed that the filter material significantly affects elemental quantification, particularly when the collected PM_10_ mass is limited, highlighting the importance of careful filter selection and handling procedures. Recommendations for optimal analytical practices are provided to minimize contamination risks and enhance reliability in trace element analysis of PM_10_ emissions. These findings contribute to refining analytical methodologies essential for accurate environmental monitoring and regulatory assessments of vehicular non-exhaust emissions.

## 1. Introduction

Atmospheric particulate matter (PM) remains a major public-health and environmental concern, and in recent decades, there has been growing evidence of the role of non-exhaust traffic emissions alongside tailpipe sources [1,2,3]. Recent reviews have shown that road-traffic, non-exhaust particles are becoming a dominant fraction of urban PM in many cities and, because of their specific size and chemical composition, contribute to adverse cardiovascular and respiratory outcomes [4,5,6]. Among non-exhaust contributors, brake-wear particles are noteworthy for their complex metal signatures (e.g., Ba, Cu, Fe, Ti, Zn, Zr) and for their potential toxicological relevance in urban environments [7,8,9,10]. As brake-wear particles (BWPs) are mainly inorganic, trace-element analysis is essential to reveal markers suitable for source apportionment of traffic-related PM. Literature reviews identified some gaps in the areas of generation, detection, quantification and characterization of airborne brake-wear emissions [1,11]. The most critical issue related to these studies is the lack of standardized procedures. Another important gap is to find the best available techniques for sampling, quantitative measurement and characterization of brake-wear particulate emissions. The chemical characterization of the PM emitted by different combinations of brake pads and discs not only allows us to clearly and immediately assess their potential influence on environmental and health issues, but also to trace the different sources of atmospheric particulate matter through the easier recognition of distinctive analyte patterns [2]. Reliable quantification of these elements is therefore essential for exposure assessment and mitigation strategy development.

Various instrumental techniques are employed to analyze the chemical composition of these emissions, each offering unique advantages: X-ray fluorescence (XRF), which enables rapid bulk elemental screening; scanning electron microscopy (SEM) for studying morphology; and X-Ray Diffraction (XRD) and Raman spectroscopy to identify molecular/phase information [12,13,14,15,16,17,18,19,20,21].

In particular, inductively coupled plasma–optical emission spectroscopy and mass spectrometry (ICP-OES, ICP-MS) have gained attention for their ability to provide a detailed elemental analysis of brake-wear particles. ICP-OES and ICP-MS are highly sensitive and capable of detecting major, minor, trace (ICP-OES) and ultra-trace (ICP-MS) elements in complex matrices, making them a powerful tool for quantifying inorganic elements in brake-wear emissions [22,23,24]. However, the sample preparation is time-consuming, and their use is less frequent compared to XRF and SEM-EDX for BWP characterization, so far. Despite this, studies have demonstrated the effectiveness of these techniques in quantifying metal concentrations in road dust and brake-wear emissions [25,26].

The sample preparation for ICP-OES and ICP-MS analysis usually requires acid dissolution of the solid samples, for instance, by the use of microwave-assisted digestion to accelerate and optimize sample dissolution, thereby improving recovery rates for element analysis [27,28,29]. However, the correct selection of the type of acid used in the process is fundamental to obtaining accurate results. Different acid mixtures are used in the procedures for PM_10_ digestion depending on the sample composition and elements of interest. Nitric acid (HNO_3_) is widely used for this purpose. It can oxidize organic matter and most inorganic compounds, although some metals are passivated (e.g., Al, Cr, Ti) [27,30]. The combination of HNO_3_ with hydrochloric acid (HCl), especially in the proportion 1:3 (aqua regia), enhances the dissolution of metals and metal oxides [30,31]. Hydrogen peroxide (H_2_O_2_) can be used to improve digestion efficiency, particularly for samples with high organic matter content. The mixture HNO_3_:H_2_O_2_ (4:1 ratio) is the acid mixture reported in the digestion method for the determination of Pb, Cd, As and Ni in PM_10_ standardized by the European Directive 2004/107/EC (UNI EN 14902:2005) [32,33,34,35]. Sometimes the mixture HNO_3_/HF/H_3_BO_3_ is also used, though less frequently due to the hazardous nature of hydrofluoric acid [25,36,37,38]. Indeed, HF is a weak acid that behaves as a strong complexing agent. It forms stable fluorides and fluorine complexes with many elements, enabling the dissolution of refractory species (e.g., silicates). H_3_BO_3_ (boric acid) is used to reduce the possible presence of excess HF in solution by forming HBF_3_OH.

Overall, ICP-OES and ICP-MS provide highly accurate elemental quantification, while XRF is preferred for rapid and non-destructive elemental analysis, and SEM-EDX is essential for morphological studies. Given these premises, the integration of several analytical techniques remains the most effective approach to gain a comprehensive understanding of brake-wear emissions and their environmental impact.

In this work, innovative acid mixtures were tested to obtain the complete dissolution of PM samples using a single acid mixture. Fluoroboric acid (HBF_4_) has emerged as a powerful alternative to hydrofluoric acid (HF) in the acid digestion of siliceous and refractory materials. The replacement of HF is highly desirable due to its acute toxicity, volatility and corrosiveness, as well as its tendency to produce insoluble metal fluorides (e.g., CaF_2_, MgF_2_, AlF_3_, Rare Earth Element fluorides), which can impair elemental recoveries and damage analytical instrumentation. HBF_4_ circumvents many of these issues by acting as a moderated source of fluoride ions, undergoing stepwise hydrolysis in aqueous solution [39].

These steps ensure the progressive release of fluoride under acidic conditions. Unlike pure HF, which often requires post-digestion neutralization with boric acid to prevent SiF_4_ volatilization or metal–fluoride precipitation, HBF_4_ offers an intrinsically buffered medium (due to the presence of boron–oxygen), especially when combined with HNO_3_ [40,41,42].

In terms of analytical performance, several studies have demonstrated that HBF4-based digestion methods can match or even exceed HF-based methods, especially for multi-elemental analysis of silicon-rich or mineral matrices such as soil, sediments, or particulate matter [40,42,43].

While optimized acid mixtures are decisive for the complete dissolution and recovery of the analytes, the substrate onto which particles are collected is equally determinative for achieving accurate results. The recent protocol “Minimum Specifications for Measuring and Characterizing Brake Emissions” edited by the Particle Measurement Program Informal Working Group (Task Force 2—Brake dust Sampling and Measurement) in July 2021 recommended PTFE-coated glass microfiber (EMFAB) and PTFE membrane filters with polymer support as a filter material for brake-wear PM collection in the context of brake homologation tests carried out by the dynamometer bench test, specifically to gravimetrically measure brake particle emissions [44]. EMFAB filters consist of borosilicate glass microfibers reinforced with woven glass cloth and bonded with PTFE, and offer greater mechanical robustness and airflow for gravimetry and handling, but their glass fiber composition can contribute to alkali/alkaline-earth background or interact with aggressive acid digests, so the control of the background contribution is more critical [45].

PTFE filters are chemically inert and consistently show the lowest elemental blanks, which is why they are preferred for trace-element determinations; but the main practical issue is related to the polymethylpentene (PMP) ring that supports the filter, and this can complicate the sectioning and digestion processes [46,47].

From these insights, our study systematically evaluates EMFAB and PTFE filters for trace-element analysis of brake-wear PM_10_ generated on a tribometer system. Specifically, we (i) compare alternative acid digestion mixtures, (ii) quantify and compare blank contributions and handling issues for different filter substrates, (iii) evaluate different calibration and drift-correction strategies for ICP-based measurements and (iv) apply the optimized workflow to PM_10_ generated by a tribometer using two friction material classes (Low-Steel, LS, and Non-Asbestos Organic, NAO). The goal is to deliver practical recommendations on filter selection and analysis (digestion and calibration) protocols that enhance data reliability in trace-element analysis of non-exhaust particulate emissions.

## 2. Results

### 2.1. Sample Pretreatment Results: Evaluation of Digestion Mixtures

**Figure 1:**
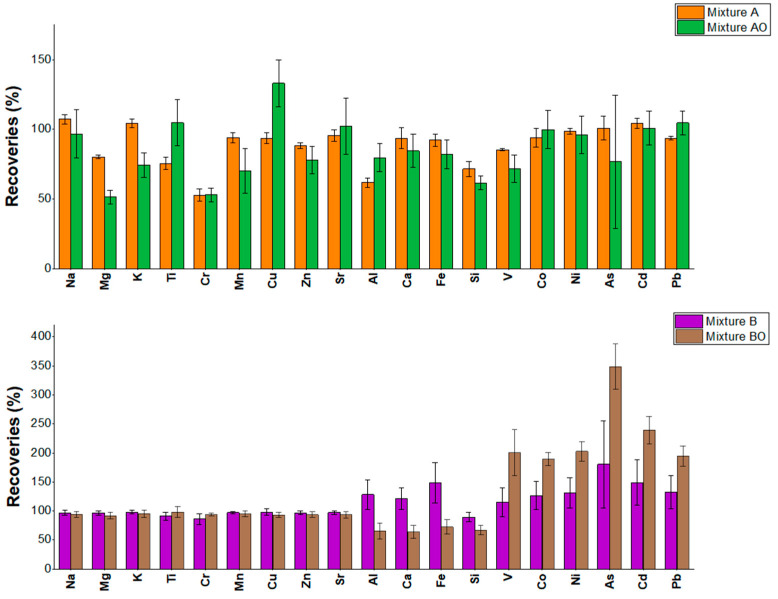
Mean values of the recoveries determined for elements by analysis of SRM NIST 1648a using extraction mixtures A (HNO_3_ + HBF_4_) and B (HNO_3_ + HBF_4_ + H_2_O_2_) in two different modes (AO and BO with a contact time of 16 h) (top: A, AO; bottom: B, BO). Black bars indicate the standard deviations.

The average recoveries, expressed as mass percentages, obtained for the nineteen elements, with their standard deviations from the analysis of Standard Reference Material NIST 1648a [48] and with the four tested digestion conditions (mixture A: 4 mL HNO_3_ + 2 mL HBF_4_; mixture AO: mixture A left in contact with the SRM for 16 h before microwave digestion; mixture B: 4 mL HNO_3_ + 1 mL HBF_4_ + 1 mL H_2_O_2_; mixture BO: mixture B left in contact with the SRM for 16 h before microwave digestion) are shown in Figure 1. Mass percentages were calculated as the mass of each analyte measured divided by the mass of that analyte in the weighted SRM.

Comparing mixture A (HNO_3_ + HBF_3_) and mixture AO (A Overnight), a paired-sample *t*-test (α = 0.05) found no significant differences, except for Si (*p*-value = 0.025), which had a higher value with A (70%) than with AO (59%). Conversely, for mixture B (HNO_3_ + HBF_3_ + H_2_O_2_) and BO (B Overnight), the recoveries differed significantly for Al, Ca, Fe, Si, V, Co, Ni, As, Cd and Pb. Al, Ca, Fe and Si were higher (*p*-values in Supplementary Table S2) with B (respectively, 142%, 130%, 165%, 92%) than with BO (respectively, 74%, 73%, 83%, 71%). For V, Co, Ni, As, Cd and Pb, recoveries were generally higher with BO (200%, 189%, 202%, 349%, 239%, 194%, respectively) than with B (115%, 127%, 131%, 180%, 149%, 133%). Recoveries above 115% (see Supplementary Table S1 for acceptance criteria) flag suspect results that may arise from the mixture and the 16 h contact time and/or ICP-MS interferences. Considering the average of the relative standard deviations (RSD%) of the elements’ recoveries per mixture, we noticed a 3% for mixture A in contrast with 12% with AO and 22% for B in respect to mixture BO (24%). Therefore, we excluded the overnight conditions from the suitable choice of attack digestion for PM_10_ samples.

To compare mixtures A, B and C (4 mL HNO_3_ + 1 mL H_2_O_2_), Table 1 reports the mean recoveries alongside AOAC acceptance ranges (which depend on the SRM analyte concentration) and Horwitz ratios for repeatability (HorRatr, as defined in Equation (2), in Section 3) values, with an acceptance range of 0.5–2 [49].

Mixture A places 13/19 analytes within the AOAC acceptance window, with four clear fails below 80% (notably, Ti 76%, Cr 53%, Al 62% and Si 72%). The precision is consistently acceptable: HorRatr falls within 0.5–2 for 15/19 analytes, with only Ca, Si, Cr exceeding two and V below the lower limit. By contrast, mixture B shows a systematic positive bias: only 10/19 analytes lie in the 80–115% window, while many exceed 125%—e.g., Co 127%, Al 128%, Ni 131%, Pb 133%, Fe 148%, Cd 149% and As 180%. This inflation in B is mirrored by poor repeatability: only 7/19 HorRatr values are in the range 0.5–2. Most fall in the 5–11 range, with a few borderline cases around 2.4–2.8 (Ti, Cr, Co). Finally, mixture C exhibited 9/19 analytes with acceptable recoveries, the lowest result among all mixtures. At least six elements reached values below 50% (K 47%, Na 44%, Al 39%, Cr 33%, Ti 19% and Si 1%). Considering repeatability, mixture C performs better than B with 12/19 elements in the 0.5–2 acceptable values.

For elements with multiple isotopes, no significant differences emerged among isotopes, except for Sr. With mixture A, ^86^Sr, ^87^Sr and ^88^Sr yielded 94%, 167% and 67%, respectively. The high ^87^Sr (7.0% natural abundance) is plausibly due to ^87^Rb (27.8% abundance) isobaric overlap, consistent with the presence of Rb in NIST 1648a (Rb at 51.0 mg/kg; Sr at 215 mg/kg). A further contribution from ^40^Ar^47^Ti^+^ is also possible, given the Ti content (4021 mg/kg; ^47^Ti abundance 7.4%) in NIST 1648a. The under-recovery of ^88^Sr is harder to rationalize, considering the possibility of the polyatomic interference ^40^Ar^48^Ti (^48^Ti abundance 73.7%), and could be attributed to an instrumental bias. The only isotope consistent with the Sr ICP-OES result is ^86^Sr.

In summary, mixture A (4 mL HNO_3_ + 2 mL HBF_4_) provides more consistent precision and adequate accuracy for most elements. In addition, HBF_4_ is confirmed to increase the solubilization of refractory elements, such as Ti, K, Al and Na, that could be occluded in the silicate fraction, and it also increased the solubilization of Cr and Si itself.

The percentage recoveries obtained using the acid mixtures A and A_modified_ (mixture A with the addition of 2 mL of HPW) and two different heating programs in a microwave oven are shown in Supplementary Figure S1. We observe a statistically significant difference (paired samples *t*-test) for Mg, K, Ti, Cu, Al and Si. Only for Ti and Al did the addition of 2 mL of HPW have a positive effect (105%, 80% and 76%, 62% as percentage recoveries for the mixtures A_modified_ and A, respectively). As could be expected, the addition of HPW dilutes the acidity and the effective concentrations of the species F^-^/HF (generated from HBF_4_), leading mainly to slowing the dissolution of silicate/oxide phases, affecting the efficiency [50]. Not only that, but the slower kinetics could probably amplify small differences in sample load, volumes and local temperature during digestion, leading to uneven degrees of dissolution across replicates, confirmed by the higher standard deviations obtained by all analytes for mixture A_modified_.

Finally, we tested mixture A on a second Standard Reference Material (BCR-176, fly ash) [51] to evaluate its applicability on samples of different matrices. Table 2 reports the mean percentage recoveries and HorRatr values.

Overall, BCR-176 shows generally acceptable accuracy and repeatability. Most targets fall in the AOAC percentage recovery window with HorRatr between 0.5 and 2. For example: Mn 90% (1.2), Fe 91% (0.9), Na 101% (1.5), Zn 88% (1.1), Ba 94% (0.9), Cu 103% (0.5), V 109% (0.6), Ni 104% (1.5), As 97% (1.9) and Pb 103% (1.0). Cr performs well with an average recovery of 78% (HorRatr: 1.2), not far from the acceptable AOAC value of 90%. In stark contrast, the use of the same mixture A in the NIST 1648a showed decreased Cr (53%) and higher HorRatr (2.1), underscoring a matrix-dependent difference in extractability. A plausible matrix-driven explanation is that, in fly ash, Cr is predominantly hosted in phases that are efficiently attacked by the HNO_3_–HBF_4_ mixture, namely the glassy aluminosilicates and Fe-oxide/spinel domains typical of combustion ash [52,53]. Under these conditions, HF/F^−^ generated from HBF_4_ dissolves silica-rich glass via hexafluorosilicate formation (e.g., [SiF_6_]^2−^), promotes the dissolution of Al-rich frameworks, and stabilizes metal–fluoro complexes (e.g., [CrF_6_]^3−^), facilitating Cr release [54]. In contrast, a larger fraction of Cr in NIST 1648a is plausibly associated with more refractory metal-rich particles, chromite-like spinels, or carbonaceous matrices that digest less completely under the same conditions; thus, recovery remains lower even though the bulk “silicate percentage” of the two SRMs is comparable (around 30%) [55]. Javed et al. (2020) and Zimmermann et al. (2020) [40,43] developed digestion methods based on mixtures that comprise nitric acid and fluoroboric acid for the analysis of soil and sediment samples. Their results yielded high recovery rates (in the range of 85–100%) for elements that are typically difficult to solubilize, including aluminium, chromium, potassium, titanium, silicon, zirconium and molybdenum (Zr and Mo silicon in the range of 85–100% (Zr and Mo could not be compared in this work because they are not present in the tested SRMs samples)) [40,43].

### 2.2. Calibration Strategy Evaluation

We also evaluated two strategies for signal drift-correction in ICP-MS: internal-standard normalization (^89^Y, ^115^In, ^125^Te, ^193^Ir) and external calibration with intermittent reads of one calibration standard following a bracketing design (EST). To test whether any internal standard provided a measurable advantage, we used a one-factor ANOVA and reported least-squares means (LS, model-adjusted means). The factor was not significant (*p* = 0.261), and the Tukey HSD post hoc test found no pairwise differences among groups; LS-means clustered tightly (^193^Ir ≈ 93.1%, ^89^Y ≈ 91.1%, EST ≈ 89.3%, ^115^In ≈ 87.5%, ^125^Te ≈ 87.1). Considering these results, we preferred to adopt the EST strategy for signal drift-correction, simplifying the workflow and avoiding potential contamination from spiking internal standards. Supplementary Figure S2 and Supplementary Table S3 show the % recoveries obtained for the isotopes by the different drift correction approaches and the outcomes of the post hoc Tukey HSD test.

### 2.3. Tribometer/Dynamometer PM_10_ Sample Comparison

To generate brake-wear PM_10_ under controlled and reproducible conditions, we used a laboratory-scale tribometer, i.e., a friction test rig in which a small pad sample is pressed against a rotating disc under controlled load and sliding speed to simulate braking events. This tribometer configuration, including the custom PM_10_ sampling chamber, is described in detail in the Materials and Methods Section 3.2.

**Figure 2:**
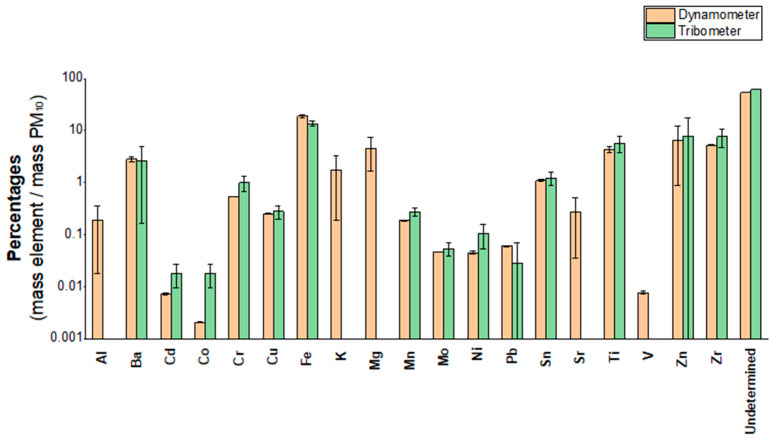
Comparison between the percentages of mass obtained by the same tribological couple tested with the EMFAB filter in the dynamometer and tribometer set-up. Undetermined is the sum of the mass of the elements not determined (C, N, S, O mainly).

As a full-scale reference, brake-wear PM_10_ was also collected on a dynamometric bench, where complete brake assemblies are operated under prescribed braking cycles and inertia conditions that are closer to real-world operation. In this section, we compare the elemental composition of PM_10_ generated by the wear of a tribological couple composed by a GCI disc and NAO pads collected on EMFAB filter on dynamometer bench (one single test performed) and the average chemical composition obtained for NET samples (five tests performed on the tribometer with EMFAB filters and the same friction material (NAO pads) and GCI disc). Figure 2 shows the percentage concentrations of the determined elements above the total mass of PM_10_ collected by the two set-ups (tribometer and dynamometer). See also Supplementary Table S4 for the exact values.

A Pearson correlation was calculated, obtaining a *p*-value < 0.0001 with a positive correlation of 0.991. While many elements showed good agreement between the two systems—including Fe, Zn, Zr, Ti, Ba and Sn for major elements but also Cu, Mn and Mo for trace elements—other elements, like K, Mg, Sr and Al, have not been quantified in tribometer tests for two different reasons: Al, Mg and Sr concentrations in the PM_10_ were overwhelmed by the sample blank, whereas K had percentage concentrations higher than 120%, probably due to some unknown contamination. Hence, these four elements are easily traceable to the filter contribution. A key difference observed between the dynamometer and the tribometer concerns the total mass of PM_10_ collected during testing. In the case of the NAO friction material, the dynamometer test, lasting approximately 36 h, allowed for the collection of a larger quantity of PM_10_, accounting for 1.65 mg, compared to an average of only 0.5 mg collected during tribometer tests after 10 h.

Overall, the data suggest a good correlation between the two set-ups, with the tribometer capable of capturing the main chemical emission profiles but also showing some degree of bias for certain elements, probably due to the high contribution of the EMFAB filter, which requires the collection of a high amount of material. This result highlights the need to investigate the use of different types of filter material.

### 2.4. Filter Blanks Comparison

**Figure 3:**
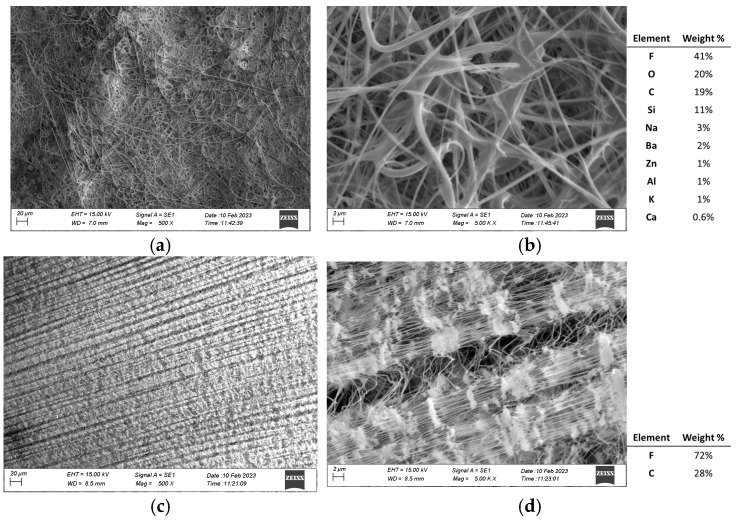
SEM images of EMFAB filter (**a**,**b**) and PTFE filter (**c**,**d**) at 500× and 5000× magnification, with their EDS spectra on the right.

Figure 3 shows the morphology of the two types of filters investigated, with their relative composition obtained by EDS spectra. The EMFAB filter reveals a chaotic, randomly oriented fibrous network. The fibers appear to be intertwined in a highly porous 3D structure, which is typical of filters made of glass wool. The random fiber arrangement and high porosity could likely facilitate particle capture by interception and impaction. The higher magnification (Figure 3b) clearly shows thin, smooth and uniform glass fibers with diameters in the sub-micron to low-micron range. This filter’s morphology is well-suited for collecting airborne particles of various sizes due to the dense yet porous fibrous mesh. The random orientation maximizes contact with particles suspended in air. EDS results highlight the complex chemical composition, confirmed also by ICP analysis (Supplementary Table S5): F and C can be traced back to the presence of the Teflon part of the EMFAB filter whereas the high presence of Si (11%), followed by Na, Ba, Zn, Al, K and Ca (summed to 9%), is associated with the presence of borosilicate glass fibers. The presence of these elements in high amounts in the filter’s background underlines the difficulty of achieving the right quantification of these analytes in the PM collected. For instance, the higher contribution of Si in the filter makes it extremely difficult to assess its quantification in PM_10_ samples and to disentangle whether the Si comes from the PM or from the filter, even after subtracting a mean value of concentration in the blank filter.

The PTFE filter surface displays a much more regular and compact structure, with a discernible grid-like or textured pattern (Figure 3c). Unlike the EMFAB filter, it lacks the fluffy, fibrous appearance and appears smoother and more compressed. At higher magnification (Figure 3d), the surface turns out to be composed of finer fibrous elements, but these fibers are embedded in a more compact, potentially layered structure. Its finer pores and smooth surface may allow for the more efficient capture of particles by diffusion. EDS spectra confirm that the composition is made of F (72%) and C (28%).

**Figure 4:**
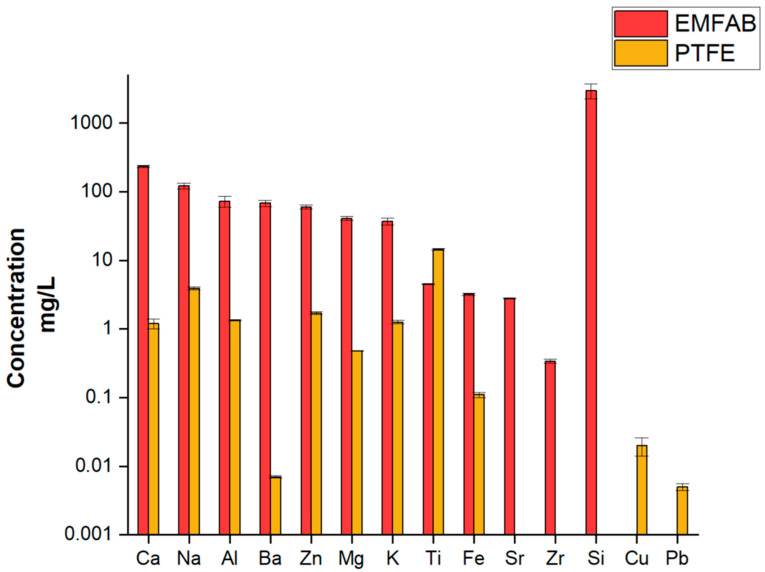
Mean concentrations of the analytes present in the digested solutions of blank EMFAB filters and PTFE filters covered with tape (three replicates). Black bars indicate standard deviations. Sr, Zr and Si are below the Limit of Detection (LOD) in PTFE filters covered with tape, whereas Cu and Pb are below the LOD in EMFAB filters.

To mitigate the risk of losing the collected PM from the PTFE filter during the PMP ring removal processes, the filters were covered with paper tape. Supplementary Figure S3 shows the concentration obtained by the digestion of a commercial paper tape. Adhesive tape showed lower concentrations and therefore appears to be the best due to the lower content of almost all the elements investigated except for Ti, which had a contribution of 0.5% of the total mass of tape digested, which could affect the right quantification of this element in the PM_10_. Despite this additional step, the concentrations of the elements present in the blank PTFE filter covered by the adhesive tape are much lower for all elements by at least one order of magnitude with respect to the EMFAB ones, except for Ti, Cu and Pb (Figure 4). For Cu, the PM_10_ concentrations in the samples were consistently about one order of magnitude higher than the corresponding PTFE blank value; in contrast, Pb was of the same order of sample concentrations, so its quantification was more susceptible to blank bias. (See Supplementary Table S6 for a comparison of the element concentrations between filter blanks and PM_10_ collected on PTFE filters).

Finally, a greater reproducibility of the results was observed with the PTFE filters covered by the tape than with the EMFAB filters. Concentration values can be seen in Supplementary Table S6.

### 2.5. PM_10_ Samples Results

**Figure 5:**
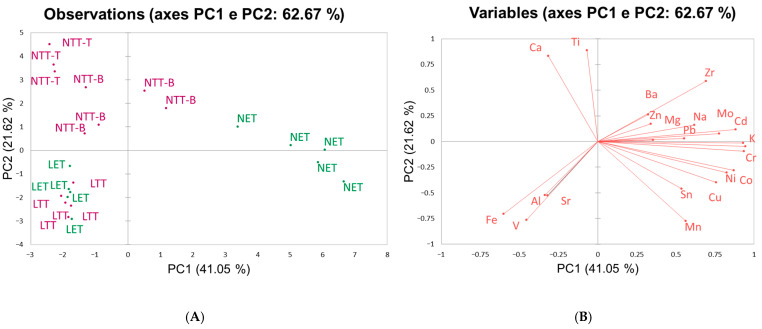
(**A**) PCA score plot of the twenty-three PM_10_ samples obtained by the tribometer set-up. In green, samples obtained with the EMFAB filter (LET for LS material, NET for NAO materials); in violet, samples obtained with the PTFE filter (LTT for LS material, NTT-T/NTT-B for NAO materials). (**B**) PCA loading plot of the twenty-three PM_10_ samples obtained by the tribometer set-up. The element concentration used for the dataset was the mass of the element on the mass of PM_10_ in percentage.

A Principal Component Analysis (PCA) was applied (Figure 5A) to the dataset composed of the elemental composition in mass percentages of the twenty-three PM_10_ samples collected on EMFAB and PTFE filters (for additional information, such as the matrix dataset, Pearson correlation matrix and eigenvalues, see Supplementary Tables S8 and S9).

The first two principal components explain 62.67% of the total variance and show a clear differentiation between the PM_10_ samples according to the type of friction material. The LS-derived sample (LET and LTT) cluster, in the lower left part of the score plot, is characterized by negative values of PC1 and PC2, while NAO-derived samples (NET and NTT) occupy mainly the upper region, characterized by positive values of PC2. A striking observation is that the difference between EMFAB (NET) and PTFE (NTT) filters appears only in NAO samples with high positive values of PC1 that grouped NET samples, whereas NTT samples are mainly characterized by negative values of PC1. Moreover, it is also possible to evidence a separation between the NTT samples with paper tape that adheres to the bottom side of the filter (NTT-B) and NTT samples with paper tape that seals the PM collected on the top side of the filter (NTT-T). This is likely due to the incomplete effectiveness of the tape placed on the bottom side of the filter in preventing partial loss of the PM during the PMP ring elimination phase. This led to a greater dispersion of the NTT-B samples and an underestimation of the element concentrations in the PM collected. Finally, the greater dispersion and separation for NAO samples can be explained by the very low mass of collected PM_10_ (always below 1 mg for the thirteen PM_10_ samples obtained by NAO brake pads wear), making the analysis more sensitive to variability and potentially less accurate due to the greater influence of the chemical composition of the filter itself and of the analytical preparation.

LS samples (LET and LTT) cluster tightly regardless of the filter type, indicating that the filter material does not significantly affect the results when PM mass is sufficiently high (7–16 mg). This suggests that for low-mass samples, such as those from NAO materials, the choice of the filter is critical for obtaining reliable compositional data, while for LS samples, the higher PM mass reduces the influence of the filter type.

The loading plot (Figure 5B) clarifies how the two components are characterized by the variables. PC1 is driven positively by Cu, Ni, Co, Cd, Cr, Mn, Sn and K and negatively by Fe, V and, to a lesser extent, Al and Sr. PC2 separates Ca, Ti, Zr and Ba (together with mildly positive Na, Mg, Zn, Pb and Mo) on the positive side from Mn–Cu–Ni–Co on the negative side. Accordingly, NET samples with high positive PC1 scores align with the Cu–Ni–Co–Cr–Mn vector, whereas the negative PC1 scores of LS-samples point toward the Fe–V direction. The positive PC2 values of most NAO samples are consistent with Ca-Ti-Zr-Ba-rich formulations (typical barite, calcium carbonate, potassium titanate, zirconium oxides/silicates), while the negative PC2 values of LS samples reflect the stronger Fe, Al and V signature expected for Low-Steel materials (due to alumina, metallic Fe, steel) [1,56].

Applying Hierarchical Cluster Analysis (Supplementary Figure S4), the whole of the information of the dataset is considered. Two clusters, one composed of NET samples and the other composed of LS (LET and LTT) and NTT samples, respectively, are obtained. At a lower dissimilarity level, LET and LTT separate from NTT samples, forming two distinct sub-clusters. Again, NET and NTT samples show a higher dissimilarity, being at the extremes of the dendrogram, underlining the greater influence of the type of filter in NAO samples.

**Figure 6:**
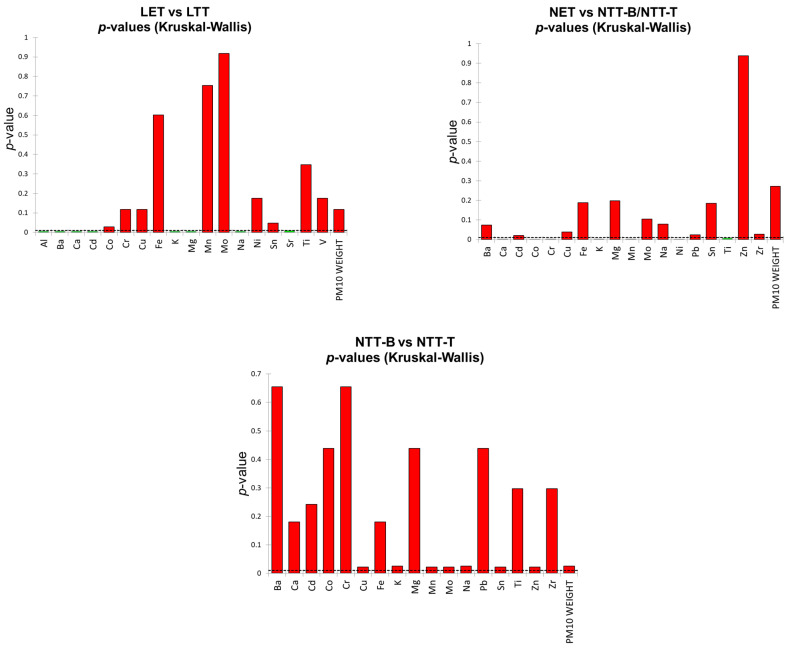
Kruskal–Wallis *p*-value graphs for group 1 (LET vs. LTT, top-left), group 2 (NET vs. NTT-B/NTT-T, top-right), and group 3 (NTT-B vs. NTT-T, bottom-middle). Dashed lines indicate significance threshold at the 99% confidence level (α= 0.01).

To further investigate whether the filter material (EMFAB vs. PTFE) or the different analytical procedure (sealing the PM_10_ with tape on the bottom or on the top of the filter) influences the chemical composition of the PM_10_ samples, a non-parametric Kruskal–Wallis test was applied to three different groups of samples: (1) LET and LTT; (2) NET and NTT; (3) NTT-T and NTT-B. This statistical approach was chosen due to the small sample size of the groups and the large number of variables considered. A significance level of α = 0.01 was selected intentionally to minimize the risk of committing a Type I error, thereby increasing the statistical stringency in detecting true differences among the groups. The *p*-values obtained for each element considering the three aforementioned groups are shown in Figure 6. The Kruskal–Wallis results for LET and LTT samples reveal that several elements exhibit statistically significant differences depending on the types of filter. Notably, Al, Ba, Ca, Cd, K, Mg, Na and Sr show *p*-values below 0.01, indicating strong evidence that their concentrations vary depending on the filter material. Except for Cd, all these elements are found in the EMFAB filter.

For the second group tested (NET vs. NTT), Ca, Co, Cr, K, Mn, Ni and Ti show a statistically significant difference between the two types of filter. In this case we find elements, such as Ca and K, that were already differentiated between the two different filter types. The difference found for Ti could be traced back to its presence in the tape used for PTFE filters. Although this difference was not emerged in group 1, it must be remembered that the samples obtained from the NAO pad wear were characterized by very low mass concentrations, and this can affect the result in different ways. Importantly, the total mass of PM_10_ collected in the two different groups tested did not differ significantly between the EMFAB and PTFE filters, indicating that the observed chemical differences are not due to systematic differences in the mass concentration collected. Finally, the NTT-T and NTT-B samples do not show statistically significant differences at the selected significance level, suggesting that the sealing process itself does not introduce a different chemical bias in the elemental distribution in PM collected on PTFE filters. The difference between these two groups can be seen at a higher significance level (α ≥ 0.05).

To assess both the analytical precision and the repeatability of the elemental concentration measurements, intra-sample and inter-sample relative standard deviations (RSD%) were calculated for each sample category. For each filter, the sample was physically divided into two halves, and each half was analyzed separately. The intra-sample RSD% was calculated for each element based on the two replicates. Finally, an overall RSD% value for each category (filter, brake pad and with tape on bottom or top) was obtained by averaging the RSD% values obtained as intra-sample. This metric reflects the sampling homogeneity of the tribometric set-up and analytical procedure applied to a single sample. The second type of RSD%, defined as inter-sample RSD%, quantifies the repeatability across different samples within the same category. For each element, the RSD% was calculated using the concentration values obtained from each filter belonging to the same experimental group. Subsequently, a global RSD% value for the category was obtained by averaging the RSD% values of all the determined elements. This second approach highlights the natural variability among different filters and represents the overall consistency of the PM_10_ collection and analysis process within each category.

The intra- and inter-sample RSD% values calculated for each experimental category are summarized in Table 3.

The intra-sample RSD% shows a better sampling homogeneity for LS filters (8% and 7% for LET and LTT, respectively). For NAO groups, an intra-sample RSD% of 10% was obtained by the NET group. The NTT groups show a lower sampling homogeneity. In general, PTFE filters were more manipulated by removing the PMP support ring, and this has more impact when the quantity of PM to analyze is very low. Precision seems to improve slightly by sealing the PM_10_ with tape on the top (15% NTT-T) with respect to doing so on the bottom (18% NTT-B). The inter-sample RSD% revealed a broader range of values, from 20% (LTT) to 50% (NET). The highest inter-sample variability in NET samples may be attributed to the lower total PM_10_ mass collected. On the other hand, LS samples (LET and LTT) exhibited the lowest inter-filter RSD%, confirming the overall stability and repeatability of the PM_10_ composition. Interestingly, the sealed NTT-T samples also showed reduced inter-filter variability (24%) compared to the NTT-B samples (41%).

## 3. Materials and Methods

### 3.1. PM_10_ Tribometer Samples

**Figure 7:**
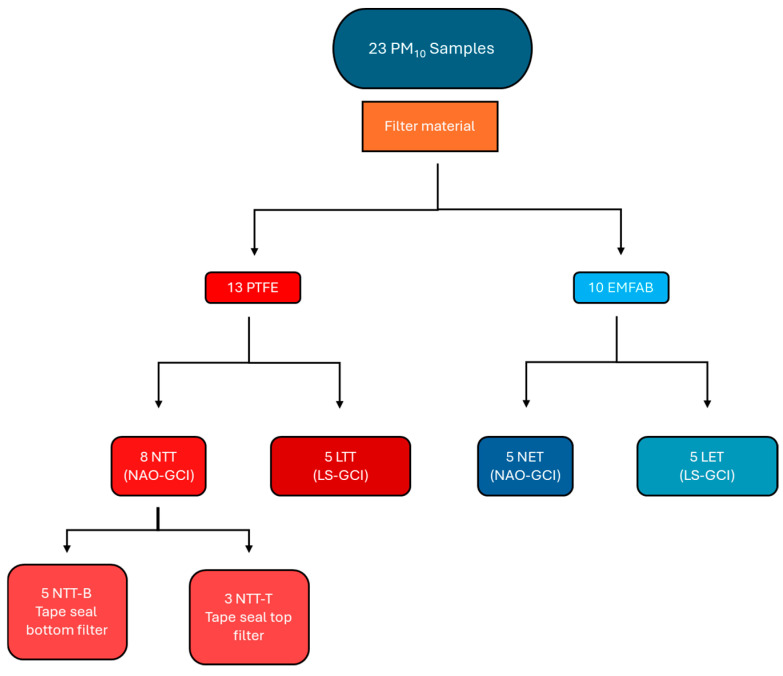
PM_10_ samples analyzed according to the different types of friction material and filter substrate.

Twenty-three PM_10_ samples were analyzed (Figure 7).

Ten samples have been obtained using a Low-Steel (LS) friction material tested with a gray cast iron disc (GCI), one half collected with the EMFAB filter (named as LET) and the other half with the PTFE filter (named as LTT). Thirteen samples were obtained using Non-Asbestos Organic (NAO) friction material tested with GCI discs, five with the EMFAB filter (NET) and eight with the PTFE filter (NTT). Of the eight NTT samples, five were with paper tape that adhered to the bottom side of the filter (NTT-B) whereas three were with paper tape that sealed the PM collected on the top side of the filter (NTT-T).

Sample name, type of friction material, type of filter, and PM_10_ mass collected are shown in Table 4.

### 3.2. Tribometer Set-Up

The tribometer model used was a Bruker UMT-TriboLab (Billerica, MA, USA). This device is composed of two main parts: a rotor in the lower part, which simulates the rotating disc of the brake system, and a carriage, free to move in the *Z*-axis direction, on which the brake pad samples reside and most of the sensors are mounted.

The brake pad samples are fixed inside a sample holder: three samples of brake pad materials are needed in order to have a stable support and to better distribute the pressure. The brake pad samples are obtained directly from a finished brake pad: the friction material is mechanically detached from the pad backplate, the thickness is set to 7.5 mm with a flat-surface grinding machine and lastly samples with a 1 cm by 1 cm area are created using a cutting machine. Tribometer discs are directly obtained from a Heavy Duty truck GCI, cut with a final diameter of 9.5 cm. Thanks to this device it is possible to define a brake procedure made up of several brakes defined by set values of sliding speed, brake time, deceleration and load force. The acquisition rate at which the instrument measures each quantity is 10 Hz. A Worldwide Harmonized Light Vehicles Test Procedure (WLTP) simulation on the tribometer was developed to replicate the official WLTP used on a chassis brake dynamometer, adapting it to the different scale and working principle. The procedure consisted of 303 individual braking events divided into 10 consecutive trips characterized by increasing average speeds. Between the individual trips, a stationary time (approx. 10 min) is required to cool the braking system and reach an ambient temperature. The total duration of the test is 10 h.

Although full-scale brake dynamometer benches remain the reference standard for regulatory testing and detailed performance evaluation, one significant disadvantage of dynamometer set-ups involves their cost and the length of the tests, whereas laboratory-scale tribometer tests are more economical and less time consuming. Given its reduced test duration and simplified set-up, the tribometer offers a practical and time-efficient solution for early-stage testing and preliminary selection of friction material pairs and filter selection, before proceeding to the reference dynamometer test.

**Figure 8:**
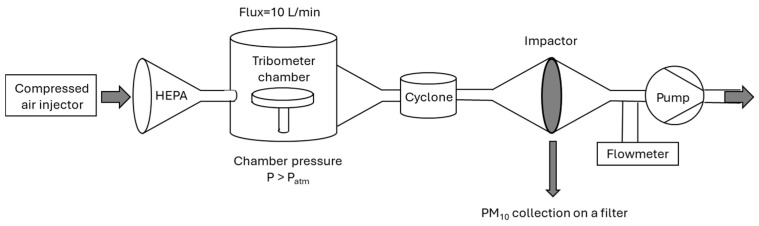
Schematic representation of PM_10_ tribometer set-up used in this work.

For the quantification and inorganic characterization of PM_10_, a stainless-steel chamber was designed to be able to convey the particulate matter emitted by the brake system during the WLTP simulation tests onto a filter. The set-up developed for the collection of the PM_10_ fraction is schematically represented in Figure 8.

The compressed air injector serves to produce a greater inlet flow so as to create a slight overpressure inside the chamber. The air, before entering the chamber, is purified through a HEPA Capsule Versapor™ (Anaheim, CA, USA) filter that guarantees 99.97% retention of particulate matter with an equivalent aerodynamic diameter of 0.3 μm. The purified air flow passes into the chamber where the wear of the friction material occurs; PM of various sizes is formed and conveyed outside the chamber to the Dekati cyclone (Kangasala, Finland). The latter is made entirely of stainless steel and is used to remove large particles (>10 µm) from an aerosol stream before the sample reaches the sampling filter. The cyclone is manufactured according to EPA standard 201A [57]. The fraction of PM_10_ selected by the cyclone passes through the filter holder and settles on the selected filter. The air flow is conveyed to the dehumidifier, and the flow rate can be controlled through a flow meter. The entire system is connected to a rotary pump (10 L/min), which enables the air flow to transport the particulate matter and convey the PM_10_ fraction to the sampling filter. Tests were conducted on 47 mm diameter EMFAB and 47 mm diameter PTFE filters. Each filter was weighed before and after the test using an analytical balance (readability 0.1 mg), and the gravimetric mass was obtained by difference.

### 3.3. Sampling Filter Blanks

In the evaluation of the suitable filter for PM_10_ collection, the inorganic chemical composition of the different types of filters was considered. Three EMFAB and three PTFE (Houston, TX, USA) filters were cut in two and analyzed with the mixture chosen according to the procedure described below. The average values of the concentrations of the filters were subtracted from the concentration of the samples, in order to eliminate the contribution of the filter. PTFE filters have a PMP (polymethylpentene) ring, which is incompatible with the acid digestion procedure because of the highly exothermic reaction of this material with the selected acid attack mixture, and the uncontrollable overpressure of the system with the risk of vessel explosion. To resolve this situation, it was decided to remove the PMP support before proceeding with the digestion of the samples. However, removing the PMP support proved to be a complicated task due to the risk of losing collected material during the manipulation. It was concluded that the best solution was to adhere the filter to a paper tape base. This solution effectively immobilized the PTFE filter mesh, preventing it from wrinkling during cutting and ensuring more accurate sample preparation. To obtain reliable concentration values for the sample blank, the PTFE filter stuck together with the paper tape was analyzed.

### 3.4. Reagents

Reagents were all of analytical purity. Water was purified in a Milli-Q system, resulting in high-purity water (HPW) with a resistivity of 18.2 MΩ∙cm. Intermediate metal standard solutions were prepared from concentrated (1000 mg/L) stock solutions (CPI International) and acidified to pH = 1.5 with nitric acid. HNO_3_ ≥ 65%, HBF_4_ 48%, were purchased from Sigma-Aldrich (Saint Louis, MO, USA), and H_2_O_2_ 30% from VWR Chemicals (Radnor, PA, USA).

### 3.5. Sample Pretreatment

Three acid mixtures were tested in this work: one consisting of 4 mL HNO_3_ and 2 mL HBF_4_ (mixture A); the second one consisting of 4 mL HNO_3_, 1 mL HBF_4_ and 1 mL H_2_O_2_ (mixture B); and the last one composed of 4 mL of HNO_3_ and 1 mL of H_2_O_2_ (mixture C) Furthermore, to assess the effect of increased contact time, additional tests were carried out where mixtures A and B remained in contact with the certified sample for 16 h before microwave digestion (AO, BO).

To evaluate the efficiency of the extraction procedure, it was necessary to evaluate the recoveries of the analytes chosen through the analysis of a standard reference material (SRM). The standard reference material 1648a (Urban Particulate Matter) was used. Once the best mixture was chosen, another standard reference material, namely BCR 176 (Fly Ash), was studied. Al, Ca, Cd, Co, Cr, Cu, Fe, K, Mg, Mn, Na, Ni, Pb, Si, Sr, Ti, V and Zn were the certified elements present and determined in SRM NIST 1648a, whereas Ba, Cr, Cu, Fe, Mn, Na, Ni, Pb and Zn were the analytes determined in SRM BCR 176.

Fifteen replicates made by 25 mg of SRM NIST 1648a were weighed. Three aliquots were treated with each type of mixture and type of contact (A, AO, B, BO, C).

The average concentrations obtained for each analyte were compared with the certified and reference values by evaluating the recovery percentages (Equation (1)). In this way, it was possible to determine the best acid mixture for each element.

(1) $$\%Recovery=\left(\frac{C}{C_{SRM}}\right)\times 100\;$$

where *C* is the concentration of the analyte in the solution after digestion, and *C_SRM_* is the concentration of the analyte in the SRM.

Acid digestion was performed in a microwave oven (power control), Milestone MLS-1200 (Fremont, CA, USA), equipped with PTFE closed-vessels. The digestion program is reported in Supplementary Table S10.

Once the program was finished, the vessels were allowed to cool under the hood before being opened. The samples were then filtered through cellulose filters (Whatman n° 5, Maidstone, UK) and placed in HDPE bottles. The solution was then brought to a volume of 25 mL by adding HPW.

An additional test was performed using the Milestone Ethos One microwave oven (equipped with a temperature control system). Triplicate digestions of NIST SRM 1648a were performed with mixture A modified with the addition of two mL of HPW to meet the instruments’ minimum total volume. The temperature ramp applied is reported in Supplementary Table S11.

Each filter was cut into two parts, and each part was digested separately, with mixture A obtaining the best recovery results.

### 3.6. Chemical Analysis

A PerkinElmer Optima 7000 DV (Waltham, MA, USA) ICP-OES was used for the quantification of major and trace elements in PM_10_ and consists of a plasma source powered by a solid-state radio-frequency generator, an Echelle monochromator and a CCD array detector (Dublin, OH, USA). The nebulization system used consists of a glass concentric nebulizer and a glass cyclonic spray chamber.

Agilent 7500ce (Santa Clara, CA, USA) ICP-MS was used for the quantification of trace and ultra-trace elements and consists of a plasma source powered by a solid-state radio-frequency generator, an interface consisting of nickel sampling and skimmer cones, a lens system, a quadrupole mass analyzer, and a secondary electron multiplier as a detector. The nebulization system consists of a glass concentric nebulizer and a quartz Scott spray chamber.

The wavelengths and isotopes used for the elements’ quantification are summarized in Supplementary Table S12.

**Figure 9:**
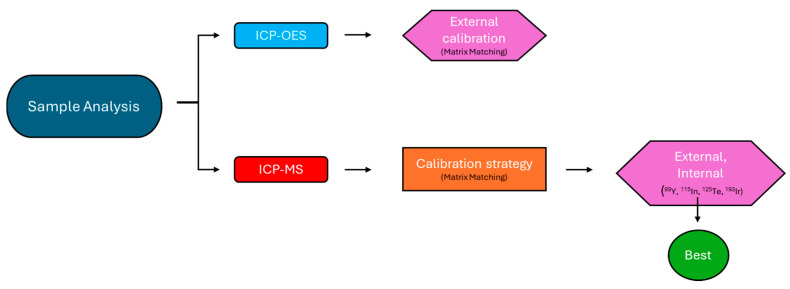
Sample analysis workflow.

Concentrations of the analytes of interest were determined with external calibration, using the Matrix Matching method. This was achieved by preparing the standard solutions using as a diluent solution one that was obtained with the same reagents used in the sample pre-treatment procedure and the same manipulation steps. To correct the instrumental drifts (i.e., the stability of the instrumental response over time), one of the standard solutions was analyzed at regular intervals over time during the instrumental analysis. Moreover, for the ICP-MS analysis, we also evaluated the use of four internal standards (^89^Y, ^115^In, ^125^Te, ^193^Ir). Figure 9 shows a resume of the workflow.

Al, As, Ba, Ca, Cd, Co, Cr, Cu, Fe, K, Mg, Mn, Mo, Na, Ni, Pb, Sn, Sr, Ti, V, Zn, and Zr were the twenty-two analytes determined in the PM_10_ samples.

Taking into account the level of element concentrations in standard reference materials and PM_10_ samples, Al, Ba, Ca, Cr, Fe, K, Mg, Mn, Na, Sn, Ti, Zn and Zr were determined by ICP-OES, and As, Cd, Co, Cr, Ni and V were quantified by ICP-MS, while Cu, Sr and Pb were quantified by both instruments. For trace/ultra-trace determinations by ICP-MS, we therefore report the result using the most abundant interference-free isotope.

For both ICP-MS and ICP-OES, the limits of detection (LOD) and the limits of quantification (LOQ) were calculated. To achieve that, a calibration curve was produced for each instrument in the concentration range of the expected LOD and LOQ values. A set of twelve procedure blanks were analyzed as samples. The LOD value for each analyte was extrapolated by dividing the standard deviation of the instrumental response of the procedural blank solutions by the slope of the calibration curve and multiplying this value by a factor of 3. Similarly, the LOQ value for each analyte was extrapolated by dividing the standard deviation of the instrumental response of the procedural blanks by the slope of the calibration curve and multiplying it by a factor of 10. The LOD and LOQ values for the major and minor elements are shown in Supplementary Tables S13 and S14.

### 3.7. SEM Analysis

To broaden the understanding of the chemico-physical characteristics of the material constituting the two types of filters selected for testing, a verification of their morphology was conducted using SEM-EDS. SEM analyses were performed with a ZEISS EVO50 XVP (Oberkochen, Germany) microscope equipped with an OXFORD X-STREAM (Ann Arbor, MI, USA) detector for X-ray microanalysis.

### 3.8. Statistical Processing

Acceptable recovery for the analytes in SRMs was evaluated considering the AOAC Guidelines, which are used here as general guidance for single-laboratory method performance, in line with the Eurachem “Fitness for Purpose of Analytical Methods” guide [48,58]. Supplementary Table S1 reports recovery limits for the analytes in SRMs as functions of the concentration. To evaluate the acceptance repeatability of the results, the Horwitz Ratio (HorRat_r_) was calculated for each analyte:

(2) $${HorRat}_{r}=\frac{{RSD}_{r}experimental}{{RSD}_{r}theoretical}$$

where RSD_r_ experimental is the relative standard deviation calculated for each analyte on the SRM replicates, and RSDr theoretical is the relative standard deviation calculated by the formula RSDr = C^−0.15^, where C is the concentration as a mass fraction of the element in the SRMs [59]. Acceptable HorRat_r_ values are in the range 0.5–2.

Results relating to the PM_10_ samples were processed by one-way ANOVA, Principal Component Analysis (PCA) and Hierarchical Clustering Analysis (HCA), using XlStat 2025 software package, an add-on for Microsoft Excel. Information on the principles of these techniques can be found elsewhere [60,61]. The non-parametric Kruskal–Wallis test was used to compare the different groups of samples to highlight if there is a statistical difference between the elements analyzed in the two types of filters. Elements that were found <LOD in both groups tested were excluded from the statistical treatment.

## 4. Conclusions

This study proposes an analytical workflow for multi-element determination in brake-wear PM_10_, from filter selection and microwave digestion to data treatment.

The mixture with 4 mL HNO_3_ + 2 mL HBF_4_ provided the best compromise between accuracy and precision, yielding AOAC-compliant recoveries and HorRat_r_ values for most elements in two SRMs, providing the expected advantage of fluoride chemistry in dissolving silicate-rich matrices.

For ICP-MS, external calibration with intermittent standard reads was adopted as a simple and reliable strategy for drift correction, which, in our work, performed comparably to internal standard calibration. PTFE filters (used with the selected low-blank paper adhesive tape) provided overall lower amount and more reproducible blanks than EMFAB ones.

Multivariate analysis applied to tribometer-generated PM_10_ samples separated samples primarily by friction class. LS aerosols formed compact clusters largely insensitive to filter type, consistent with higher collected masses (7–16 mg). NAO aerosols, by contrast, were low-mass and more filter-sensitive; this underscores the need for PTFE substrates and a strict blank control when analyzing low PM_10_ mass.

Because brake-wear PM is predominantly inorganic, obtaining reliable measurements depends on the use of substrates with minimal blanks and on a digestion mixture that quantitatively allows the release of elements from silicate-rich matrices. Overall, the proposed workflow enables trace- and ultra-trace-level element determinations with reproducible performance, which is essential to discriminate brake-derived PM from other urban particulate matter sources and to generate comparable, decision-grade data for monitoring and mitigation of non-exhaust emissions.

## Figures and Tables

**Table 1:** Mean recoveries and HorRatr values of the analytes determined in SRM NIST 1648a between mixtures A, B and C. Values in bold indicate the acceptable values according to AOAC guidelines [48].

Analyte	A Mean Recoveries (%)	A HorRatr	B Mean Recoveries (%)	B HorRatr	C Mean Recoveries (%)	C HorRatr
Na	**107**	**1.0**	**97**	**1.4**	44	8.0
Mg	80	**0.8**	**97**	**1.9**	77	**1.0**
K	**104**	**1.4**	**98**	**1.8**	47	10.6
Ti	76	**1.9**	**91**	2.5	19	3.6
Cr	53	2.1	**86**	2.8	33	4.3
Mn	**94**	**1.2**	**97**	**0.6**	**92**	**0.5**
Cu	**94**	**1.4**	**98**	**1.8**	**93**	**1.2**
Zn	88	**0.8**	**97**	**1.1**	**91**	**1.8**
Sr	**96**	**1.1**	**97**	**0.8**	72	**0.5**
Al	62	**1.8**	128	6.6	39	8.5
Ca	**94**	4.0	121	7.5	**94**	**0.9**
Fe	**92**	**1.6**	148	7.8	81	**1.1**
Si	72	3.8	89	4.6	1	64.0
V	**85**	0.2	**115**	3.6	76	**1.5**
Co	**94**	**0.9**	127	2.4	**79**	**0.5**
Ni	**98**	**0.5**	131	5.0	**89**	**0.6**
As	**101**	**1.4**	180	7.0	**102**	0.4
Cd	**104**	**0.6**	149	4.4	**97**	**0.5**
Pb	**94**	**0.5**	133	7.1	**91**	**0.9**

**Table 2:** Mean percentage recoveries and HorRatr values of the elements analyzed in BCR 176. Values in bold indicate the acceptable values according to AOAC guidelines [48].

Analyte	Mean Recoveries (%)	HorRatr
Mn	**90**	**1.2**
Fe	91	**0.9**
Na	**101**	**1.5**
Zn	88	**1.1**
Ba	**94**	**0.9**
Cr	78	**1.2**
Cu	**103**	**0.5**
V	**109**	**0.6**
Ni	**104**	**1.5**
As	**97**	**1.9**
Pb	**103**	**1.0**

**Table 3:** Inter-sample and intra-sample RSD% values for the five types of samples analyzed.

Group	Intra-Sample RSD%	Inter-Sample RSD%
NET	10	50
NTT-B	18	41
NTT-T	15	24
LET	8	22
LTT	7	20

**Table 4:** Sample ID for the twenty-three samples analyzed, with the indication of type of the filter, type of the friction material and PM_10_ weight (mg) obtained.

Sample ID	Type of Friction Material	Type of Filter	PM_10_ Weight (mg)
NTT-B 26	NAO	PTFE	0.46
NTT-B 43	NAO	PTFE	0.57
NTT-B 54	NAO	PTFE	0.80
NTT-B 55	NAO	PTFE	0.28
NTT-B 56	NAO	PTFE	0.44
NTT-T 80	NAO	PTFE	0.16
NTT-T 81	NAO	PTFE	0.15
NTT-T 82	NAO	PTFE	0.14
NET 14	NAO	EMFAB	0.56
NET 16	NAO	EMFAB	0.56
NET 41	NAO	EMFAB	0.47
NET 47	NAO	EMFAB	0.23
NET 42	NAO	EMFAB	0.44
LTT 28	LS	PTFE	7.09
LTT 37	LS	PTFE	12.52
LTT 38	LS	PTFE	7.47
LTT 40	LS	PTFE	13.93
LTT 49	LS	PTFE	8.05
LET 17	LS	EMFAB	17.34
LET 33	LS	EMFAB	8.96
LET 34	LS	EMFAB	10.64
LET 35	LS	EMFAB	13.26
LET 36	LS	EMFAB	16.34

## Data Availability

Data available on request.

## Supplementary Materials

The following supporting information can be downloaded at: https://www.mdpi.com/article/10.3390/molecules30244816/s1.

## References

[1] Amato F. (2018). Non-Exhaust Emissions: An Urban Air Quality Problem for Public Health: Impact and Mitigation Measures.

[2] OECD (2020). Non-Exhaust Particulate Emissions from Road Transport: An Ignored Environmental Policy Challenge.

[3] Piscitello A., Bianco C., Casasso A., Sethi R. (2021). Non-exhaust traffic emissions: Sources, characterization, and mitigation measures. Sci. Total Environ..

[4] Oroumiyeh F., Jerrett M., Del Rosario I., Lipsitt J., Liu J., Paulson S.E., Ritz B., Schauer J.J., Shafer M.M., Shen J. (2022). Elemental composition of fine and coarse particles across the greater Los Angeles area: Spatial variation and contributing sources. Environ. Pollut..

[5] Grigoratos T., Martini G. (2014). Brake Wear Particle Emissions: A Review. Environ. Sci. Pollut. Res..

[6] Fussell J.C., Franklin M., Green D.C., Gustafsson M., Harrison R.M., Hicks W., Kelly F.J., Kishta F., Miller M.R., Mudway I.S. (2022). A Review of Road Traffic-Derived Non-Exhaust Particles: Emissions, Physicochemical Characteristics, Health Risks, and Mitigation Measures. Environ. Sci. Technol..

[7] Sanders P.G., Xu N., Dalka T.M., Maricq M.M. (2003). Airborne brake wear debris: Size distributions, composition, and a comparison of dynamometer and vehicle tests. Environ. Sci. Technol..

[8] Gietl J.K., Lawrence R., Thorpe A.J., Harrison R.M. (2010). Identification of brake wear particles and derivation of a quantitative tracer for brake dust at a major road. Atmos. Environ..

[9] Stojanovic N., Glisovic J., Abdullah O.I., Belhocine A., Grujic I. (2022). Particle formation due to brake wear, influence on human health and measures for their reduction: A review. Environ. Sci. Pollut. Res..

[10] Paithankar J.G., Saini S., Dwivedi S., Sharma A., Chowdhuri D.K. (2021). Heavy metal associated health hazards: An interplay of oxidative stress and signal transduction. Chemosphere.

[11] Kukutschová J., Filip P., Amato F. (2018). Chapter 6—Review of brake wear emissions: A review of brake emission measurement studies: Identification of gaps and future needs. Non-Exhaust Emissions.

[12] Philippe F., Morgeneyer M., Xiang M., Manokaran M., Berthelot B., Chen Y.-M., Charles P., Guingand F., Bressot C. (2021). Representativeness of airborne brake wear emission for the automotive industry: A review. Proc. Inst. Mech. Eng. Part D J. Automob. Eng..

[13] Hagino H. (2024). Feasibility of Measuring Brake-Wear Particle Emissions from a Regenerative-Friction Brake Coordination System via Dynamometer Testing. Atmosphere.

[14] Tsyupa B., Bandiera M., Federici M., Leonardi M. (2022). Comparative study of size distribution and chemical composition of emissions from low-steel and NAO friction materials. EuroBrake 2022—Technical Content.

[15] Kadachi A.N., Al-Eshaikh M.A. (2012). Limits of detection in XRF spectroscopy. X-Ray Spectrom..

[16] Newbury D.E., Ritchie N.W.M. (2013). Is scanning electron microscopy/energy dispersive X-ray spectrometry (SEM/EDS) quantitative?. Scanning.

[17] Bachchhav B.D., Hendre K.N. (2022). Wear performance of asbestos-free brake pad materials. Jordan J. Mech. Ind. Eng..

[18] Löber M., Bondorf L., Grein T., Reiland S., Wieser S., Epple F., Philipps F., Schripp T. (2024). Investigations of airborne tire and brake wear particles using a novel vehicle design. Environ. Sci. Pollut. Res..

[19] Woo S.-H., Lee G., Han B., Lee S.-H. (2022). Development of dust collectors to reduce brake wear PM emissions. Atmosphere.

[20] Zhang K., Xu Z., Rosenkranz A., Song Y., Xue T., Fang F. (2019). Surface- and tip-enhanced Raman scattering in tribology and lubricant detection—A prospective. Lubricants.

[21] Bernardini S., Bellatreccia F., Casanova Municchia A., Della Ventura G., Sodo A. (2019). Raman spectra of natural manganese oxides. J. Raman Spectrosc..

[22] Lee E.S. (2023). Tracer-gas-integrated measurements of brake-wear particulate matter emissions from heavy-duty vehicles. Environ. Sci. Technol..

[23] Matchett L.C., Abou-Ghanem M., Stix K.A.R., McGrath D.T., Styler S.A. (2022). Ozone uptake by commercial brake pads and brake pad components: Assessing the potential indirect air quality impacts of non-exhaust emissions. Environ. Sci. Atmos..

[24] Neukirchen C., Saraji-Bozorgzad M.R., Mäder M., Mudan A.P., Czasch P., Becker J., Di Bucchianico S., Trapp C., Zimmermann R., Adam T. (2024). Comprehensive elemental and physical characterization of vehicle brake wear emissions from two different brake pads following the global technical regulation methodology. SSRN.

[25] Conca E., Malandrino M., Diana A., Abollino O., Giacomino A., Bartrolí R., Moreno T., Querol X., Amato F. (2022). Methods for elemental analysis of size-resolved PM samples collected on aluminium foils: Results of an intercomparison exercise. Molecules.

[26] Adamiec E., Jarosz-Krzemińska E., Wieszała R. (2016). Heavy metals from non-exhaust vehicle emissions in urban and motorway road dusts. Environ. Monit. Assess..

[27] Bussan D.D. (2024). An environmentally compatible and less costly (greener) microwave digestion method of bone samples using dilute nitric acid for analysis by ICP-MS. Res. Sq..

[28] Al-Hakkani M.F. (2019). Guideline of inductively coupled plasma mass spectrometry (ICP-MS): Fundamentals, practices, determination of the limits, quality control, and method validation parameters. SN Appl. Sci..

[29] Aldabe J., Santamaría C., Elustondo D., Lasheras E., Santamaría J.M. (2013). Application of microwave digestion and ICP-MS to simultaneous analysis of major and trace elements in aerosol samples collected on quartz filters. Anal. Methods.

[30] Papadopoulos A., Assimomytis N., Varvaresou A. (2022). Sample preparation of cosmetic products for the determination of heavy metals. Cosmetics.

[31] Moursy A.R., Ahmed N., Sahoo R. (2020). Determination of total content of some microelements in soil using two digestion methods. Int. J. Chem. Stud..

[32] (2005). Qualità Dell’aria Ambiente—Metodo Normalizzato per la Misurazione di Pb, Cd, As e Ni nella Frazione PM10 del Particolato in Sospensione.

[33] Kayaba S., Kajino M. (2023). Potential impacts of energy and vehicle transformation through 2050 on oxidative-stress-inducing PM2.5 metals concentration in Japan. GeoHealth.

[34] Bukowiecki N., Lienemann P., Hill M., Figi R., Richard A., Furger M., Rickers K., Falkenberg G., Zhao Y., Cliff S.S. (2009). Real-world emission factors for antimony and other brake wear related trace elements: Size-segregated values for light- and heavy-duty vehicles. Environ. Sci. Technol..

[35] Diana A., Bertinetti S., Abollino O., Giacomino A., Buoso S., Favilli L., Inaudi P., Malandrino M. (2022). PM10 element distribution and environmental-sanitary risk analysis in two Italian industrial cities. Atmosphere.

[36] Yadav A.K., Bhowmik D., Sikder N. (2012). Direct determination of zirconium and silicon in zircon by flame atomic absorption spectrometry using two rapid decomposition methods. Anal. Methods.

[37] Camilleri R., Stark C., Vella A.J., Harrison R.M., Aquilina N.J. (2023). Validation of an optimised microwave-assisted acid digestion method for trace and ultra-trace elements in indoor PM2.5 by ICP-MS analysis. Heliyon.

[38] Lough G.C., Schauer J.J., Park J.-S., Shafer M.M., DeMinter J.T., Weinstein J.P. (2005). Emissions of metals associated with motor vehicle roadways. Environ. Sci. Technol..

[39] Wamser C.A. (1948). Hydrolysis of fluoboric acid in aqueous solution. J. Am. Chem. Soc..

[40] Javed M.B., Grant-Weaver I., Shotyk W. (2020). An optimized HNO3 and HBF4 digestion method for multielemental soil and sediment analysis using inductively coupled plasma quadrupole mass spectrometry. Can. J. Soil Sci..

[41] Sucharová J., Suchara I. (2006). Determination of 36 elements in plant reference materials with different Si contents by inductively coupled plasma mass spectrometry: Comparison of microwave digestions assisted by three types of digestion mixtures. Anal. Chim. Acta.

[42] Krachler M., Mohl C., Emons H., Shotyk W. (2002). Influence of digestion procedures on the determination of rare earth elements in peat and plant samples by USN-ICP-MS. J. Anal. At. Spectrom..

[43] Zimmermann T., von der Au M., Reese A., Klein O., Hildebrandt L., Pröfrock D. (2020). Substituting HF by HBF4—An optimized digestion method for multi-elemental sediment analysis via ICP-MS/MS. Anal. Methods.

[44] PMP—Particle Measurement Programme Informal Working Group Task Force 2—Brake Dust Sampling and Measurement. Proceedings of the Minimum Specifications for Measuring and Characterizing Brake Emissions.

[45] Chow J.C., Watson J.G., Wang X., Abbasi B., Reed W.R., Parks D. (2022). Review of filters for air sampling and chemical analysis in mining workplaces. Minerals.

[46] Lee J., Ryu J.-S., Jeong S., Kim J., Jeong H., Ra K., Yang M., Chang H.J. (2021). Elemental and isotopic compositions in blank filters collecting atmospheric particulates. J. Anal. Sci. Technol..

[47] (1987). U.S. EPA. Guidelines for PM-10 Sampling and Analysis Applicable to State Implementation Plans.

[48] (2020). Urban Particulate Matter.

[49] Horwitz W. (2002). AOAC Guidelines for Single-Laboratory Validation of Chemical Methods for Dietary Supplements and Botanicals.

[50] Shi Z., Bonneville S., Krom M.D., Carslaw K.S., Jickells T.D., Baker A.R., Benning L.G. (2011). Iron dissolution kinetics of mineral dust at low pH during simulated atmospheric processing. Atmos. Chem. Phys..

[51] (1984). Certified Reference Material BCR176 Fly Ash.

[52] Vereshchak M., Manakova I., Shokanov A., Sakhiyev S. (2021). Mössbauer studies of narrow fractions of fly ash formed after combustion of Ekibastuz coal. Materials.

[53] Strzałkowska E. (2021). Morphology, chemical and mineralogical composition of magnetic fraction of coal fly ash. Int. J. Coal Geol..

[54] Mitra A., Rimstidt J.D. (2009). Solubility and dissolution rate of silica in acid fluoride solutions. Geochim. Cosmochim. Acta.

[55] Huggins F.E., Huffman G.P., Robertson J.D. (2000). Speciation of elements in NIST particulate matter SRMs 1648 and 1650. J. Hazard. Mater..

[56] Diana A., Conca E., Bartrolí R., Moreno T., Querol X., Padoan E., Abollino O., Inaudi P., Malandrino M., Amato F. (2025). Particulate matter emissions from brake pads: A comparative study of low-steel and non-asbestos organic materials. SSRN.

[57] (2020). PM10 and PM2.5—Constant Sampling Rate Procedure.

[58] Magnusson B., Örnemark U. (2014). Eurachem Guide: The Fitness for Purpose of Analytical Methods—A Laboratory Guide to Method Validation and Related Topics.

[59] Horwitz W., Albert R. (2006). The Horwitz Ratio (HorRat): A Useful Index of Method Performance with Respect to Precision. J. AOAC Int..

[60] Einax J.W., Zwanziger H.W., Geiß S. (1997). Chemometrics in Environmental Analysis.

[61] Miller J.N., Miller J.C., Miller R.D. (2018). Statistics and Chemometrics for Analytical Chemistry.

