# Supplementary material for: Evaluation of Filter Types for Trace Element Analysis in Brake Wear PM10: Analytical Challenges and Recommendations
Source: Molecules. 2025 Dec 18;30(24):4816. doi: 10.3390/molecules30244816 (PMC12736183; doi:10.3390/molecules30244816)
Supplement: Supplementary file 1 [file molecules-30-04816-s001.zip › molecules-4014910-supplementary.pdf]

Supplementary Material

Evaluation of Filter Types for Trace Element Analysis in Brake Wear PM_{10}: Analytical Challenges and Recommendations

Aleandro Diana [1,2], Mery Malandrino [1,2,*], Riccardo Cecire [1,3], Paolo Inaudi [4], Agnese Giacomino [4], Ornella Abollino [4], Agusti Sin [2,5] and Stefano Bertinetti [1,*]

1 Department of Chemistry, University of Turin, 10125 Turin, Italy; aleandro.diana@unito.it (A.D.); riccardo.cecire@unito.it (R.C.)
2 UniTo-ITT JointLab, 10135 Turin, Italy; agusti.sin@itt.com
3 Department of Agricultural, Forest and Food Sciences, University of Turin, 10095 Grugliasco, Italy
4 Department of Drug Science and Technology, University of Turin, 10125 Turin, Italy; paolo.inaudi@unito.it (P.I.); agnese.giacomino@unito.it (A.G.); ornella.abollino@unito.it (O.A.)
5 ITT Friction Technologies, 12032 Barge, Italy
* Correspondence: mery.malandrino@unito.it (M.M.); stefano.bertinetti@unito.it (S.B.)

Table S1. AOAC ranges of values for recovery of the analytes and Relative Standard Deviations (RSD) for repeatability and reproducibility in function of the concentration of the analyte to determine.

Concentration (mass fraction)	Range recovery acceptance (%)	Acceptance RSD_r (%)	Analyte ICP-OES	Analyte ICP-MS
100%	98-101	1		
10%	95-102%	1.5		
1%	92-105%	2	Ca, Si, K, Mg, Pb	
0.10%	90-108%	3	Cu, Mn, Na, Ti, Zn, Al, Fe	Pb
0.01%	85-110%	4	Cr, Sr	Cu,
10 ppm	80-115%	6		Cd, Ni, Sr, V, As
1 ppm	75-120%	8		Co
10 ppb	70-125%	15		

Table S2. p-values of the analytes obtained from t-test (α=0.05) for the recoveries (%) obtained comparing mixture A/AO; B/BO; A/B; A-$A_{modified}$. In bold, values that are statistically different.

Analyte	p-values (A-AO)	p-values (B-BO)	p-values (A-B)	p-values (A-$A_{modified}$)
Na	0.24	0.53	**0.01**	0.43
Mg	0.07	0.26	**0.01**	**0.004**
K	0.20	0.55	**0.03**	**0.02**
Ti	0.23	0.35	**0.01**	**0.01**
Cr	0.07	0.30	**0.01**	0.94
Mn	0.68	0.50	0.18	0.13
Cu	0.22	0.28	0.21	**0.002**
Zn	0.21	0.38	**0.01**	0.26
Sr	0.17	0.30	0.54	0.68
Pb	1.00	0.37	0.10	0.85
Al	0.13	**0.02**	**0.01**	**0.03**
Ca	0.10	**0.01**	**0.03**	0.22
Fe	0.15	**0.02**	**0.02**	0.09
Si	**0.02**	**0.03**	**0.01**	**0.03**
V	0.12	**0.03**	0.26	0.05
Co	0.31	**0.02**	0.06	0.65
Ni	0.07	**0.02**	0.06	0.83
As	0.15	**0.03**	0.30	0.58
Cd	0.09	**0.03**	0.28	0.75
Pb	0.08	**0.03**	0.05	0.52

Table S3. Post-Hoc Tukey test (HSD). Analysis of the difference between the five approaches with 95% confidence interval.

Pairwise comparison	Difference (estimated mean/LS-mean difference)	Standardised difference	Critical value	p-value for the differnce	Significance
^{193}Ir vs ^{125}Te	6.020	1.944	2.742	0.296	No
^{193}Ir vs ^{115}In	5.611	1.812	2.742	0.368	No
^{193}Ir vs EST	3.777	1.220	2.742	0.740	No
^{193}Ir vs ^{89}Y	2.018	0.652	2.742	0.966	No
^{89}Y vs ^{125}Te	4.002	1.308	2.742	0.686	No
^{89}Y vs ^{115}In	3.593	1.175	2.742	0.766	No
^{89}Y vs EST	1.759	0.575	2.742	0.979	No
EST vs $^{12.5}$Te	2.243	0.733	2.742	0.949	No
EST vs ^{115}In	1.834	0.600	2.742	0.975	No
^{115}In vs ^{125}Te	0.409	0.134	2.742	1.000	No

q studentized range statistic: 3.877

Minimum significant difference:8.593

Table S4. Comparison between the percentages in mass and their standard deviations obtained by the same tribological couple tested with EMFAB filter in dynamometer and tribometer set-up. Undetermined is the sum of the mass of the elements not determined (C, N, S, O mainly). At the bottom of the table is present the PM mass collected in mg (namely the total PM mass for tribometer is calculated as an average value of five tests, whereas for Dynamometer is the result of one single test).

Variable	Dynamometer	Tribometer
Fe	18.8 ± 0.9	14 ± 2
Zn	7 ± 6	8 ± 10
Zr	5.2 ± 0.2	8 ± 3
Mg	5 ± 3	-
Ti	4.3 ± 0.5	6 ± 2
Ba	3 ± 0.3	2.6 ± 2.4
K	1.7 ± 1.6	-
Sn	1.09 ± 0.04	1.2 ± 0.3
Cr	0.54 ± 0.01	1.00 ± 0.33
Sr	0.3 ± 0.2	-
Cu	0.25 ± 0.01	0.28 ± 0.08
Al	0.19 ± 0.17	-
Mn	0.185 ± 0.005	0.28 ± 0.05
Pb	0.060 ± 0.002	0.03 ± 0.04
Mo	0.0461± 0.0003	0.05 ± 0.02
Ni	0.045 ± 0.003	0.10 ± 0.05
V	0.008 ± 0.001	-
Cd	0.0071 ± 0.0003	0.02 ± 0.01
Co	0.0021 ± 0.0003	0.02 ± 0.01
Undetermined	53	61
PM_{10} weight (mg)	1.65	0.5 ± 0.1

Table S5. Average concentration values of the elements of interest present in the digested solutions of EMFAB filters obtained by three replicates.

Analyte	Concentration (mg/L)
Ca	235 ± 9
Na	122 ± 12
Al	72 ± 13
Ba	68 ± 7
Zn	60 ± 4
Mg	41 ± 3
K	37 ± 4
Ti	4.52 ± 0.09
Fe	3.2 ± 0.12
Sr	2.8 ± 0.05
Zr	0.34 ± 0.02
*Si	* 3000
Cr, Mn, Sn, V, As, Cd, Co, Cu, Mo, Ni, Pb	< LOD

*Si is a major component of the EMFAB filter matrix. Its concentration in the digested solutions was above the linear range of the routine ICP-OES calibration; the value of approximately 3000 mg L^{-1} was obtained from a dilution of 1:100 dilution and is reported here only to indicate the order of magnitude of the Si blank. Consequently, silicon was not quantified in PM_{10} samples.

Table S6. Comparison between average concentrations in mg/L of digested solutions of EMFAB filters, digested solutions of PTFE filters covered by adhesive paper and PM_{10} samples already subtracted by the PTFE filter contribution (LTT and NTT).

Analyte	PTFE blank (mg/L)	EMFAB blank (mg/L)	LTT (mg/L)	NTT (mg/L)
Al	1.34	72	1.2-3.3	<LOD
As	<LOD	<LOD	<LOD	<LOD
Ba	0.01	68	1.4-3	0.2-0.5
Ca	1.17	235	1.7-2.4	0.7-1.8
Cd	<LOD	<LOD	<LOD	0.002-0.005
Co	<LOD	<LOD	0.01-0.015	0.005-0.01
Cr	<LOD	<LOD	0.8-1.5	0.02-0.1
Cu	0.02	<LOD	0.5-0.9	0.04-0.06
Fe	0.11	3	106-209	1.5-11
K	1.26	37	0.8-1.3	0.4-0.8
Mg	0.48	41	1.9-4	0.1-0.3
Mn	<LOD	<LOD	0.5-1	0.02-0.06
Mo	<LOD	<LOD	0.04-0.07	0.01-0.1
Na	3.85	122	1-2.1	0.7-3
Ni	<LOD	<LOD	0.05-0.1	<LOD
Pb	0.01	<LOD	<LOD	<LOD
Sn	<LOD	<LOD	2.7-5.4	0.1-0.3
Sr	<LOD	3	0.07-0.1	<LOD
Ti	14.66	5	0.8-2.6	1.5-6
V	<LOD	<LOD	0.01-0.03	<LOD
Zn	1.70	60.00	<LOD	0.2-1.2
Zr	<LOD	0.34	<LOD	0.7-1

Table S7. Average concentration values of the elements of interest present in the digested solutions of PTFE filters covered by adhesive paper obtained by three replicates.

Analyte	Concentration (mg/L)
Ti	14.4 ± 0.3
Na	3.9 ± 0.2
Zn	1.70 ± 0.08
Al	1.34 ± 0.03
K	1.26 ± 0.07
Ca	1.2 ± 0.2
Mg	0.48 ± 0.008
Fe	0.11 ± 0.01
Cu	0.02 ± 0.006
Ba	0.007 ± 0.0003
Pb	0.005 ± 0.0006
Cr, Mn, Sn, V, As, Cd, Co, Zr, Mo, Ni, Si, Sr	< LOD

Table S8. Data matrix used for the principal component analysis (PCA). Elemental mass fractions in mass % of PM_{10} samples collected from the different tribological couples (NET, NTT-B, NTT-T, LTT, LET). These values were used as input variables for the PCA shown in Figure 5.

Object	Al	Ba	Ca	Cd	Co	Cr	Cu	Fe	K	Mg	Mn	Mo	Na	Ni	Pb	Sn	Sr	Ti	V	Zn	Zr
**NET 41**	0.087	0.0006	0.23	0.015	0.015	0.884	0.243	14.487	174.480	92.381	0.293	0.047	191.482	0.037	0.014	1.091	0.014	6.686	0.000	10.589	7.142
**NET 42**	0.087	10.5859	0.23	0.017	0.019	1.001	0.281	14.601	160.591	0.025	0.294	0.050	97.625	0.104	0.086	1.162	0.014	4.430	0.000	0.134	5.812
**NET 47**	0.087	0.0006	0.23	0.015	0.031	1.511	0.283	4.226	220.934	0.025	0.351	0.055	0.202	0.146	0.010	1.172	0.014	0.012	0.000	0.134	5.426
**NET 14**	0.087	0.0006	0.23	0.011	0.008	0.603	0.194	11.783	106.668	1.215	0.210	0.038	287.642	0.071	0.000	0.877	0.014	3.882	0.000	29.753	6.978
**NET 16**	0.087	0.0006	0.23	0.027	0.015	1.017	0.402	24.360	102.525	0.025	0.239	0.081	114.314	0.158	0.002	1.815	0.014	7.977	0.000	0.134	12.617
**NTT-B 26**	0.087	2.3032	5.22	0.012	0.000	0.306	0.167	21.136	4.349	0.025	0.110	0.082	7.570	0.001	0.014	1.272	0.014	31.668	0.000	2.295	5.619
**NTT-B 43**	0.087	2.1424	4.71	0.021	0.002	0.411	0.298	17.430	3.665	0.025	0.087	0.069	5.028	0.001	0.000	1.531	0.014	23.031	0.000	2.348	4.098
**NTT-B 54**	0.087	1.0384	5.61	0.0001	0.000	0.235	0.130	33.982	2.657	0.441	0.178	0.019	8.903	0.001	0.000	1.002	0.014	4.707	0.000	3.923	3.090
**NTT-B 55**	0.087	1.2904	8.01	0.0001	0.000	0.183	0.054	13.378	4.816	0.025	0.060	0.021	7.028	0.001	0.000	1.016	0.014	13.855	0.000	3.197	6.007
**NTT-B 56**	0.087	1.1975	4.08	0.0001	0.000	0.239	0.243	22.100	2.263	0.025	0.105	0.019	4.093	0.001	0.000	1.052	0.014	8.671	0.000	1.016	3.930
**NTT-T 80**	0.087	1.4272	5.48	0.0001	0.000	0.249	0.001	12.658	8.574	0.025	0.004	0.000	16.451	0.001	0.000	0.017	0.014	28.838	0.000	0.134	4.254
**NTT-T 81**	0.087	1.6334	6.18	0.0001	0.000	0.299	0.001	17.158	10.011	0.025	0.004	0.000	14.343	0.001	0.000	0.017	0.014	20.597	0.000	0.134	5.095
**NTT-T 82**	0.087	1.7844	14.44	0.0001	0.000	0.293	0.001	19.769	12.532	0.025	0.004	0.000	29.290	0.001	0.000	0.017	0.014	26.771	0.000	0.134	6.144
**LTT 28**	0.570	0.5811	0.65	0.0001	0.004	0.273	0.173	43.234	0.300	0.833	0.216	0.020	0.708	0.034	0.000	1.126	0.027	0.553	0.011	0.134	0.025
**LTT 37**	0.608	0.5127	0.41	0.0001	0.003	0.297	0.160	41.663	0.202	0.728	0.205	0.014	0.330	0.020	0.000	0.983	0.019	0.205	0.007	0.134	0.025
**LTT 38**	0.415	0.4871	0.57	0.0001	0.002	0.294	0.144	35.483	0.254	0.630	0.182	0.013	0.496	0.019	0.000	0.908	0.025	0.272	0.004	0.134	0.025
**LTT 40**	0.596	0.5155	0.43	0.0001	0.003	0.266	0.157	37.487	0.244	0.714	0.192	0.000	0.191	0.018	0.000	0.968	0.019	0.012	0.006	0.134	0.025
**LTT 49**	0.612	0.5658	0.64	0.0001	0.003	0.335	0.178	43.245	0.304	0.799	0.230	0.014	0.480	0.023	0.000	1.083	0.027	0.795	0.006	0.134	0.025
**LET 17**	0.087	0.0006	0.23	0.0001	0.004	0.222	0.153	36.484	0.058	0.025	0.171	0.014	0.202	0.027	0.000	0.861	0.185	0.215	0.006	0.134	0.025
**LET 33**	0.087	0.0006	0.23	0.0001	0.005	0.308	0.160	45.604	0.058	0.025	0.300	0.014	0.202	0.020	0.000	1.066	0.326	0.277	0.006	0.134	0.025
**LET 34**	0.087	0.0006	0.23	0.0001	0.004	0.273	0.124	35.396	0.058	0.025	0.225	0.013	0.202	0.023	0.000	0.831	0.250	0.125	0.006	0.134	0.036
**LET 35**	0.087	0.0006	0.23	0.0001	0.004	0.247	0.148	38.847	0.058	0.025	0.195	0.014	0.202	0.029	0.000	0.835	0.216	0.168	0.005	0.134	0.022
**LET 36**	0.087	0.0006	0.23	0.0001	0.004	0.199	0.100	38.447	0.058	0.025	0.133	0.015	0.202	0.035	0.000	0.600	0.140	0.007	0.000	0.134	0.019

Note: For some NET samples and for elements largely contributed by the EMFAB filter (e.g. Na, K, Ca, Mg), the apparent "mass %" values can exceed 100 %. This reflects the fact that, in low-mass NET tests, the blank contribution from the EMFAB filter can be comparable to or larger than the PM_{10} mass collected on the filter.

Table S9. Pearson correlation matrix of the 22 elemental mass fractions used in the PCA. Correlation coefficients are computed on the autoscaled variables (mean-centered and scaled to unit variance). Bold values indicate correlations significantly different from zero at $\alpha = 0.05$. Eigenvalues, explained variance (%) and cumulative variance (%) for the principal components obtained from the PCA of the elemental mass fractions are reported at the bottom of the table.

Variables	Al	Ba	Ca	Cd	Co	Cr	Cu	Fe	K	Mg	Mn	Mo	Na	Ni	Pb	Sn	Sr	Ti	V	Zn	Zr
**Al**	**1**	-0.145	-0.276	-0.325	-0.179	-0.217	-0.008	**0.567**	-0.280	-0.097	0.183	-0.305	-0.247	-0.123	-0.161	0.116	-0.168	-0.383	**0.707**	-0.191	**-0.514**
**Ba**	-0.145	**1**	0.136	0.278	0.184	0.232	0.131	-0.324	0.265	-0.117	-0.004	0.224	0.084	0.137	**0.915**	0.036	-0.286	0.230	-0.281	-0.130	0.228
**Ca**	-0.276	0.136	**1**	-0.187	**-0.471**	-0.319	**-0.548**	-0.367	-0.295	-0.150	**-0.788**	-0.174	-0.192	**-0.494**	-0.160	**-0.489**	-0.376	**0.775**	**-0.438**	-0.106	0.309
**Cd**	-0.325	0.278	-0.187	**1**	**0.633**	**0.760**	**0.786**	**-0.535**	**0.684**	0.247	0.337	**0.911**	**0.511**	**0.674**	0.398	**0.623**	-0.346	0.178	**-0.462**	0.245	**0.727**
**Co**	-0.179	0.184	**-0.471**	**0.633**	**1**	**0.960**	**0.628**	-0.405	**0.930**	0.275	**0.727**	**0.506**	0.370	**0.886**	**0.485**	0.389	-0.091	-0.347	-0.185	0.112	0.382
**Cr**	-0.217	0.232	-0.319	**0.760**	**0.960**	**1**	**0.663**	**-0.550**	**0.954**	0.287	**0.610**	**0.617**	**0.445**	**0.877**	**0.481**	0.405	-0.287	-0.149	-0.341	0.160	**0.579**
**Cu**	-0.008	0.131	**-0.548**	**0.786**	**0.628**	**0.663**	**1**	-0.104	**0.552**	0.173	**0.664**	**0.766**	0.325	**0.688**	0.321	**0.882**	-0.127	-0.320	-0.057	0.113	0.347
**Fe**	**0.567**	-0.324	-0.367	**-0.535**	-0.405	**-0.550**	-0.104	**1**	**-0.623**	-0.212	0.208	**-0.425**	**-0.448**	-0.280	-0.312	0.091	**0.578**	**-0.547**	**0.799**	-0.362	**-0.772**
**K**	-0.280	0.265	-0.295	**0.684**	**0.930**	**0.954**	**0.552**	**-0.623**	**1**	**0.452**	**0.581**	**0.533**	**0.601**	**0.787**	**0.537**	0.286	-0.307	-0.147	-0.403	0.336	**0.585**
**Mg**	-0.097	-0.117	-0.150	0.247	0.275	0.287	0.173	-0.212	**0.452**	**1**	0.272	0.170	**0.478**	0.019	0.104	0.085	-0.126	-0.035	-0.151	0.290	0.238
**Mn**	0.183	-0.004	**-0.788**	0.337	**0.727**	**0.610**	**0.664**	0.208	**0.581**	0.272	**1**	0.327	0.260	**0.647**	0.336	**0.613**	0.273	**-0.747**	0.330	0.117	-0.092
**Mo**	-0.305	0.224	-0.174	**0.911**	**0.506**	**0.617**	**0.766**	**-0.425**	**0.533**	0.170	0.327	**1**	0.360	**0.542**	0.340	**0.741**	-0.297	0.206	-0.396	0.204	**0.622**
**Na**	-0.247	0.084	-0.192	**0.511**	0.370	**0.445**	0.325	**-0.448**	**0.601**	**0.478**	0.260	0.360	**1**	**0.413**	0.246	0.136	-0.271	-0.027	-0.355	**0.859**	**0.590**
**Ni**	-0.123	0.137	**-0.494**	**0.674**	**0.886**	**0.877**	**0.688**	-0.280	**0.787**	0.019	**0.647**	**0.542**	**0.413**	**1**	0.391	**0.464**	-0.102	-0.361	-0.147	0.118	**0.476**
**Pb**	-0.161	**0.915**	-0.160	0.398	**0.485**	**0.481**	0.321	-0.312	**0.537**	0.104	0.336	0.340	0.246	0.391	**1**	0.181	-0.175	-0.018	-0.231	-0.042	0.246
**Sn**	0.116	0.036	**-0.489**	**0.623**	0.389	0.405	**0.882**	0.091	0.286	0.085	**0.613**	**0.741**	0.136	**0.464**	0.181	**1**	-0.045	-0.325	0.104	0.053	0.188
**Sr**	-0.168	-0.286	-0.376	-0.346	-0.091	-0.287	-0.127	**0.578**	-0.307	-0.126	0.273	-0.297	-0.271	-0.102	-0.175	-0.045	**1**	**-0.426**	**0.473**	-0.208	**-0.556**
**Ti**	-0.383	0.230	**0.775**	0.178	-0.347	-0.149	-0.320	**-0.547**	-0.147	-0.035	**-0.747**	0.206	-0.027	-0.361	-0.018	-0.325	**-0.426**	**1**	**-0.552**	-0.026	**0.484**
**V**	**0.707**	-0.281	**-0.438**	**-0.462**	-0.185	-0.341	-0.057	**0.799**	-0.403	-0.151	0.330	-0.396	-0.355	-0.147	-0.231	0.104	**0.473**	**-0.552**	**1**	-0.274	**-0.735**
**Zn**	-0.191	-0.130	-0.106	0.245	0.112	0.160	0.113	-0.362	0.336	0.290	0.117	0.204	**0.859**	0.118	-0.042	0.053	-0.208	-0.026	-0.274	**1**	0.338
**Zr**	**-0.514**	0.228	0.309	**0.727**	0.382	**0.579**	0.347	**-0.772**	**0.585**	0.238	-0.092	**0.622**	**0.590**	**0.476**	0.246	0.188	**-0.556**	**0.484**	**-0.735**	0.338	**1**

	PC1	PC2	PC3
Eigenvalue	9.030	4.757	2.047
Explained variance (%)	41.047	21.624	9.306
Cumulative variance (%)	41.047	62.671	71.977

Table S10. Heating program used for microwave-assisted acid digestion with Milestone MLS-1200.

Step	Power (W)	Time (min)
1	250	1
2	0	2
3	200	5
4	350	5
5	550	5
6	250	5
7	0	40

Table S11. Heating program used for tests with Ethos One microwave oven.

Step	Temperature (°C)	Time (min)
1	25-190	20
2	190	15

Table S12. Instrumental parameters used for ICP analysis.

Analyte	Wavelength (nm)	m/z
Al	396.153	
As		75
Ba	233.527	
Ca	317.933	
Cd		112
Co		59
Cr	267.716	
Cu	327.393	65
Fe	238.204	
K	766.490	
Na	589.592	
Mg	285.213	
Mn	257.610	55
Mo		98
Ni		62
Pb	220.353	208
Si	251.611	
Sn	189.927	
Sr	407.711	86
Ti	344.940	
V	292.402	51
Zn	206.200	
Zr	339.197	

Table S13. *Perkin Elmer Optima 7000 DV* ICP-OES Limit of Detection (LOD) and Limit of Quantification (LOQ) values in µg/L. LOD for solid PM_{10} are calculated considering a mass value of 25 mg of PM_{10} and are expressed as mg/g.

Analyte	LOD µg/L	LOQ µg/L	LOD mg/g
Al	60	200	0.06
Ba	0.4	1	0.0004
Ca	158	529	0.2
Cr	2	6	0.002
Fe	33	109	0.03
K	40	133	0.04
Mg	17.6	58.7	0.02
Mn	2.6	9	0.003
Na	140	467	0.1
Si	202	673	0.2
Sn	12	39	0.01
Sr	10	34	0.01
Ti	8	26	0.008
Zn	93	309	0.09
Zr	17	55	0.02

Table S14. *Agilent 7500ce* ICP-MS Limit of Detection (LOD) and Limit of Quantification (LOQ) values in µg/L. LOD for solid PM_{10} are calculated considering a mass value of 25 mg of PM_{10} and are expressed as mg/g.

Analyte	LOD µg/L	LOQ µg/L	LOD mg/g
As	4.3	13	0.004
Cd	0.1	0.4	0.0001
Co	0.3	1	0.0003
Cu	0.4	1.2	0.0004
Mo	0.02	0.07	0.00002
Ni	0.6	2	0.0006
Pb	0.3	1	0.0003
V	0.1	0.5	0.0001

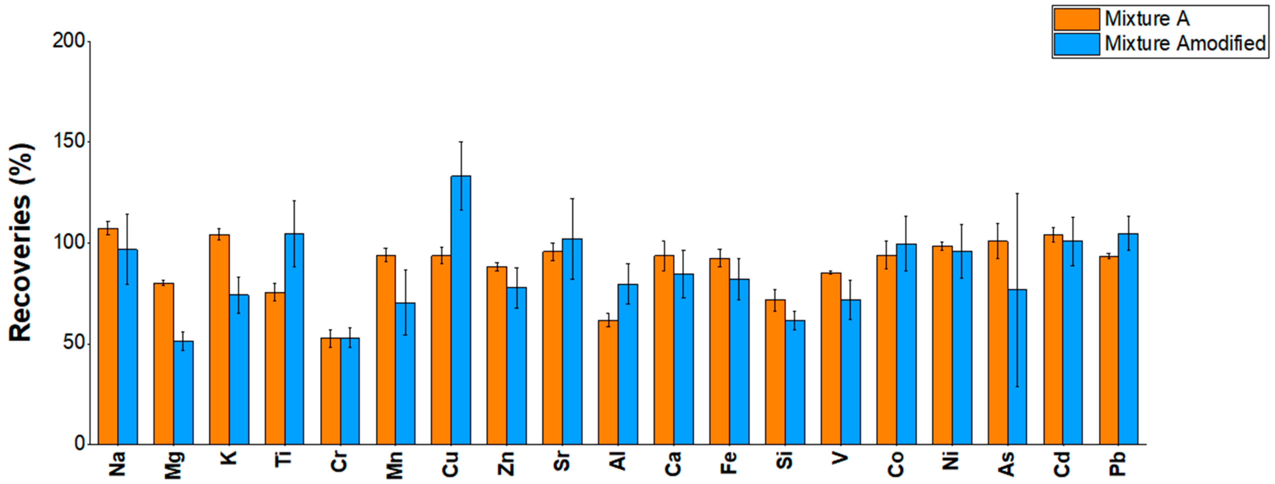

Figure S1. Mean recoveries of SRM NIST 1648a per analyte using two different microwave digestor and Mixture A and $A_{modified}$.

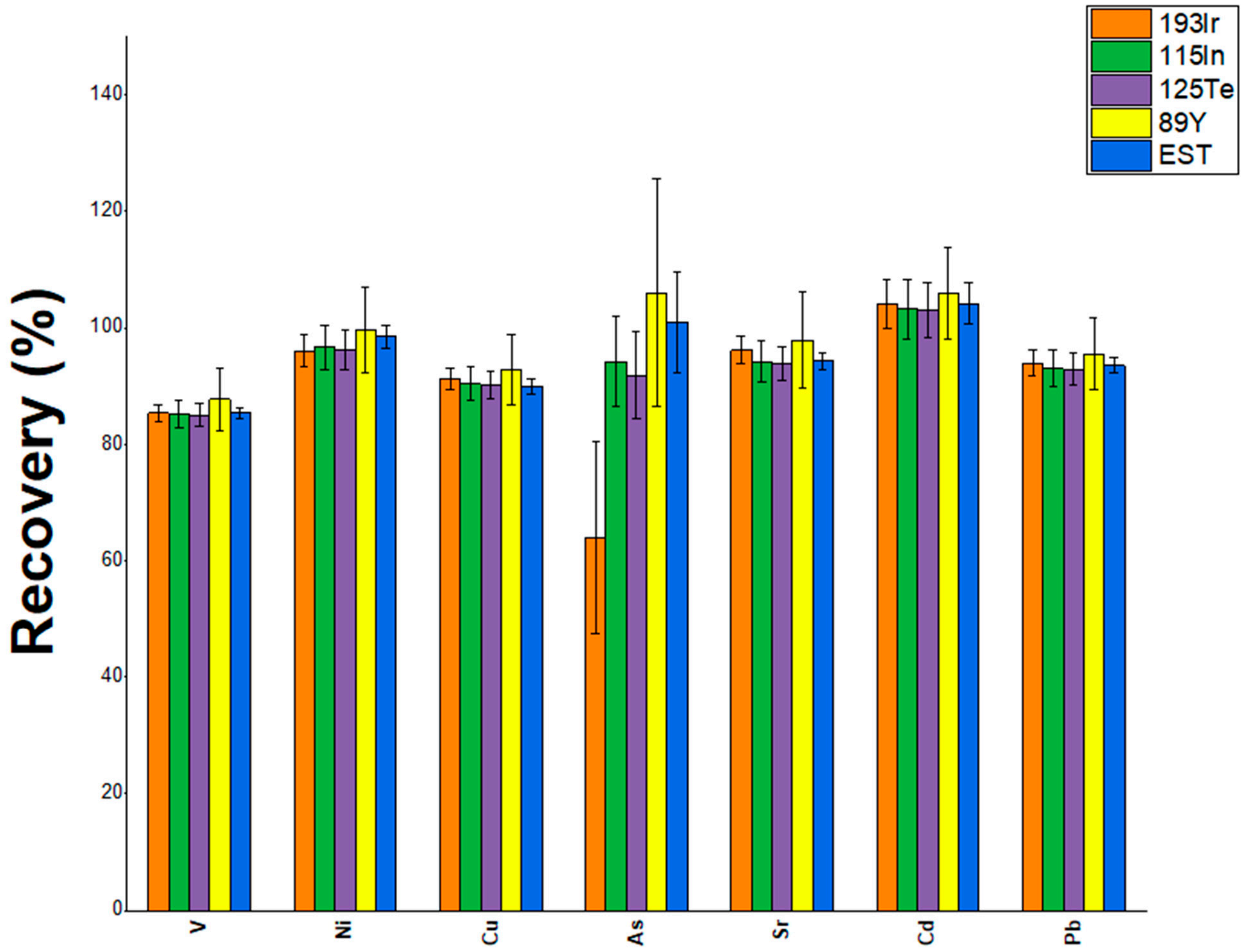

Figure S2. Mean recoveries of SRM NIST 1648a per analyte using four different internal standard and external calibration for the isotopes analysed with ICP-MS.

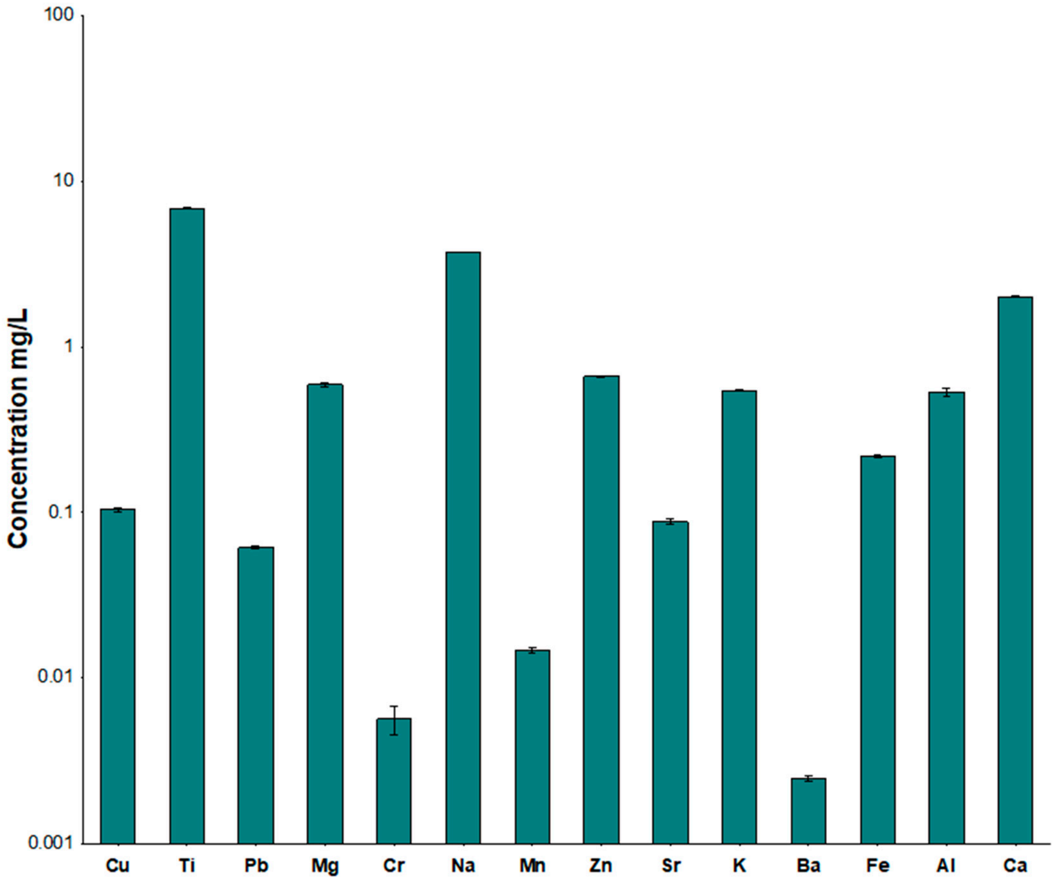

Figure S3. Average concentrations and standard deviations of the analytes present in the digested solutions of paper adhesive tape.

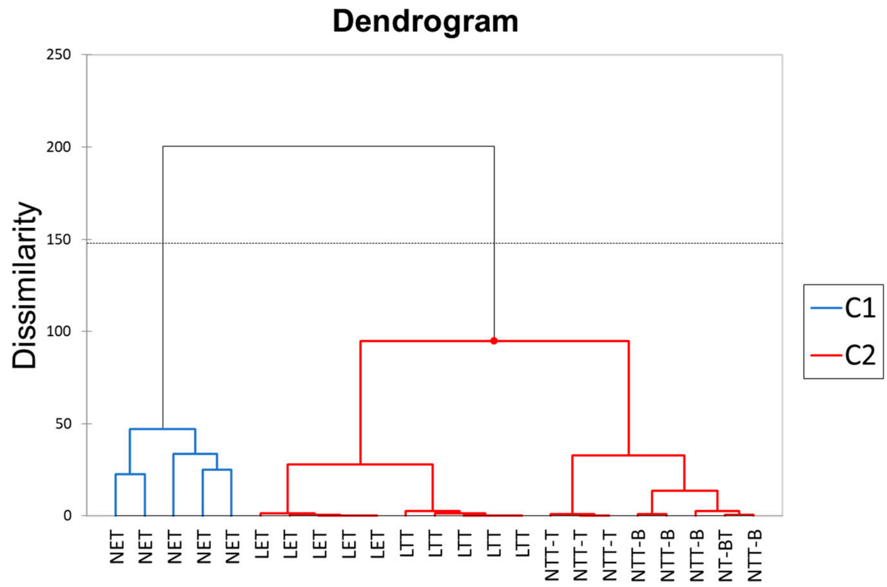

Figure S4. HCA dendrogram on the dataset of the twenty-three PM_{10} samples obtained by tribometer set-up.
